# Exploiting graphlet decomposition to explain the structure of complex networks: the GHuST framework

**DOI:** 10.1038/s41598-020-69795-1

**Published:** 2020-07-30

**Authors:** Rafael Espejo, Guillermo Mestre, Fernando Postigo, Sara Lumbreras, Andres Ramos, Tao Huang, Ettore Bompard

**Affiliations:** 1 ICAI, Instituto de Investigación Tecnológica, Universidad Pontificia Comillas, Madrid, Spain; 2 Dipartmento Energia, Politecnico di Torino, Turin, Italy

**Keywords:** Energy science and technology, Physics

## Abstract

The characterization of topology is crucial in understanding network evolution and behavior. This paper presents an innovative approach, the GHuST framework to describe complex-network topology from graphlet decomposition. This new framework exploits the local information provided by graphlets to give a global explanation of network topology. The GHuST framework is comprised of 12 metrics that analyze how 2- and 3-node graphlets shape the structure of networks. The main strengths of the GHuST framework are enhanced topological description, size independence, and computational simplicity. It allows for straight comparison among different networks disregarding their size. It also reduces the complexity of graphlet counting, since it does not use 4- and 5-node graphlets. The application of the novel framework to a large set of networks shows that it can classify networks of distinct nature based on their topological properties. To ease network classification and enhance the graphical representation of them, we reduce the 12 dimensions to their main principal components. Furthermore, the 12 dimensions are easily interpretable. This enables the connection between complex-network analyses and diverse real applications.

## Introduction

The analysis of complex-network topology can support the understanding of the principles that guide network evolution and that condition network behavior^1^. The characterization of network structure has traditionally been done through a set of global or local statistics such as degree distribution or motifs^2,3^. Both global and local metrics complement each other, since different communities may coexist in the same network with different topological properties (what is known as structural subunits)^4^. Global metrics, such as network diameter or characteristic path length, provide a panoramic view of networks that may have implications on their dynamics. For instance, the particular degree distribution of computing networks, they are scale-free networks, makes them relatively resistant to accidental failures but vulnerable to targeted attacks^5^. However, global metrics disregard the complexity of local structures that might be crucial to understand the behavior of networks, as it has been shown for the case of the internet network^6^. Furthermore, local processes condition the development of network topology^7^. Consequently, topological analyses should include the use of local statistics that zoom in the local structure of complex networks.

An example of a local-topological statistic is the motif distribution. Motifs are recurring subgraphs patterns that appear more often in a given network than in a random one. Motifs were proposed to understand the evolutionary design principles of complex networks from a local perspective^8^. They search for key local structures that determine network behavior. However, the choice of the null model (random networks to which a network is compared) to detect motifs in real networks may be misleading^9^. Furthermore, motifs are partial subgraphs (they do not necessarily include all the connections between a set of considered nodes); this leads to a loss of information that may be compelling to understand network structure^10^.

**Figure 1:**
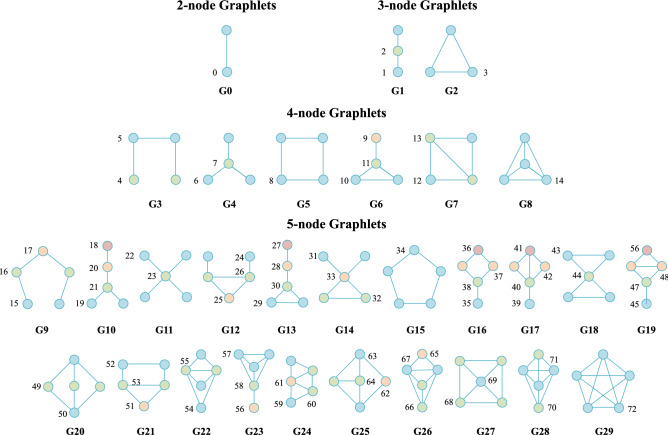
2- to 5-node graphlets (from ${G}_{0}$ to ${G}_{29}$) and their automorphism orbits (from 0 to 72). For each graphlet, nodes in the same automorphism orbits are identified with the same color (e.g. all blue nodes in ${G}_{1}$ are in ${O}_{1}$, they are in a symmetric position in the graphlet, the green node is in a different topological position, it is in ${O}_{2}$).

Unlike motifs, graphlets allow for network decomposition in small subgraphs that preserve all connections among nodes. Graphlets are small connected induced subgraphs of a large network^11^. The presence of graphlets in a network is not conditioned by a null model; they can appear at any frequency. This is a strength with respect to motifs studies. Although graphlets may be comprised of an arbitrary number of nodes, the most commonly studied graphlets are 2- to 5-node subgraphs, given that higher degrees entail higher computational complexity. The automorphism orbit of a graphlet is defined as the set of nodes that are topologically symmetric in the graphlet^12^. Orbits, therefore, define the relative position of nodes with respect to the rest of the nodes in the graphlets. Figure 1 shows all 2- to 5-node graphlets and their automorphism orbits. Finally, the description of network topology is limited by graphlet size. Although larger graphlets may complete the description of network topology, this would be unmanageable from a computational point of view. Recent works have proposed efficient algorithms for graphlet counting^13–17^.

Several models developed for the network alignment problem prove the adequacy of graphlet as a local topology descriptor^18–21^. The network alignment problem aims to find corresponding nodes between different networks. Nodes that play a similar role in both networks from a topological point of view. In this field, graphlet decomposition has been revealed as a crucial tool with a view to solving the problem. The basis of those models is the degree signature of a graphlet^12^. The graphlet degree signature is an extension of the node degree that quantifies the number of times a node in the network appears in an orbit (referred to as “touch an orbit”). Consequently, graphlets provide a complete description of local network topology (the orbits each node touches) that enhances the solution of network alignment problem. Similarly, graphlets might support the comparison among networks or the study of the role played by nodes in the network^22,23^. Despite being a good descriptor of local properties, the use of graphlet distribution (or graphlet degree signature) is not enough to have an insight into the global topological properties of networks. Yaveroğlu et al. propose the analysis of orbit correlation to characterize network structure and to ease the interpretation and implications of topological properties in real applications^24^.

This paper improves the characterization and understanding of network topology by proposing the GHuST framework that extends graphlet analysis. The advantages of this novel method are enhanced topological description, size independence, and computationally simplicity. First, the 12 dimensions fully describe the structure of networks, covering the most relevant aspects of local and global topology from a systematic manner. Second, the GHuST framework explains network properties regardless of network size. This supports the comparison among networks with different number of nodes and edges. Third, it only considers 2-node and 3-node graphlets and they follow easily from the adjacency matrix. It reduces computational complexity with respect to prior analyses that require counting higher-node graphlets.

The application of the method to a set of five real networks demonstrates the accuracy of the framework to explain network topology. Furthermore, this new metric enhances network classification and can be used as a tool to confirm the topological accuracy of synthetic networks. This validation is usually missing in the generation of synthetic power grids, where there is a weak topological validation or it is done only by a few global statistics^25^. Therefore, this tool can be introduced to compare the topology of both real and synthetic networks in a systematic manner.

The rest of the paper is organized as follows: “Understanding network structure from local properties” section presents the GHuST framework. “Explaining the topology of real networks” section illustrates its application to explain the topological structure of networks from different nature. “A panoramic view offered by local properties” section uses dimensionality reduction methods to evaluate the performance of the proposed metric when applied to a large sample of networks. Finally, “Conclusion” section presents paper conclusions.

## Understanding network structure from local properties

As explained above, graphlets can be a convenient tool for explaining the local structure of networks. Unfortunately, graphlet decomposition does not consider any interaction between graphlets. In addition, in large networks, counting graphlets is computationally intensive. It also supplies a substantial number of dimensions that are difficult to interpret (30 graphlets and 73 orbits in the case of using from 2- to 5-node graphlets). Motivated by this desire to simplify and improve topological analyses through graphlet decomposition, this section proposes a novel method that reduces the topological analysis of networks to a 12-dimensional metric, the GHuST framework. This metric can be calculated in any non-directed and unweighted network.

The 12 dimensions are obtained from the decomposition of networks in 2-node and 3-node graphlets, comprising three graphlets (${G}_{0},{G}_{1}$ and ${G}_{2}$) and four orbits (${O}_{0}$, ${O}_{1}$, ${O}_{2}$, ${O}_{3}$). The adjacency matrix succinctly reveals the number of times a node touches those orbits (see Supplementary Information Sect. 1). As explained in the prior section, recent works focused on counting graphlets efficiently in large networks^13–17^.

In addition, for the four orbits, ${P}_{t,i}$ is a binary variable that is 1 if node $i$ is at least once in orbit $t$ or 0 otherwise (1).

1 $${P}_{t,i}=\left\{\begin{array}{c}1, {O}_{t,i}>0\\ 0, {O}_{t,i}=0\end{array}\right.$$

To enhance readability, the 12 dimensions are classified into four categories: Global connectivity, Hubs, Strings, and Triangles. Those categories cover different aspects of network structure that might condition network behavior. Furthermore, these categories allow for an intuitive interpretation of topology implications in real-world applications. For instance, in power networks, the higher presence of strings might mean a lower level of network robustness (higher probability of having energy not supplied in the network in case of line failure, given that when there is a failure in a string all the downstream nodes will be affected). Similarly, the presence of large strings in an email graph (nodes stands for community members and edges connect the people who send an email with the people who receive the email) will show that the community may follow a clearly defined hierarchical structure.

To enhance network comparison, it is desirable that the 12 dimensions of the metric range between 0 and 1. In cases where a dimension does not do it, we propose a scaling factor. The 12 dimensions are defined as follows.

### Global connectivity

#### Line-surplus coefficient, ${{{\rho}}}_{1}$

It stands for the surplus of lines in the network with respect to the minimum number of lines needed to build a connected graph (2). Given a set of nodes, $N$, the minimum number of lines, ${L}_{0}$, to have a connected graph is ${L}_{0}=N-1$, in case of large networks ${L}_{0}\approx N$. As we only consider connected graphs, $N=\sum_{i}{P}_{0,i}$. The number of lines installed in a network is $\frac{\sum_{i}{O}_{0,i}}{2}$. This dimension is therefore related to the average node degree and it supplies information about line density in a network. In networks with a radial structure (trees), ${\rho {^{\prime}}}_{1}$ tends to zero. The higher the value of ${\rho {^{\prime}}}_{1}$ the more meshed a network is.

2 $${\rho {^{\prime}}}_{1}= \frac{1}{2} \frac{\sum_{i}{O}_{0,i}}{\sum_{i}{P}_{0,i}}-1$$

We define ${\rho }_{1}$ (3) to scale ${\rho {^{\prime}}}_{1}$ between 0 and 1. Networks with ${\rho }_{1}$ close to 1 have a highly meshed structure.

${\rho }_{1}$ can be rewritten as (4).

3 $${\rho }_{1}=1-\frac{1}{{\rho {^{\prime}}}_{1}+1}$$

4 $${\rho }_{1}=1- \frac{2 \sum_{i}{P}_{0,i} }{\sum_{i}{O}_{0,i}}$$

#### Leaf rate, ${{{\rho}}}_{2}$

This ratio compares the proportion of nodes with just one connection, known as leaf nodes, to the rest of nodes in the network that are not vertices of a triangle. This ratio discerns between networks in which edges may form a homogenous mesh that touches most nodes and networks characterized by the presence of hubs connecting low-degree nodes. This metric is calculated as the complementary of the ratio between the number of nodes that touches ${O}_{1}$ but does not touch ${O}_{3}$ and the number of nodes that touches ${O}_{2}$ but does not touch ${O}_{3}$ (5).

All sets of three-connected nodes are either in graphlets ${G}_{1}$ or ${G}_{2}$. For those nodes that belong to ${G}_{2}$ and they are not part of ${G}_{3}$, they may touch ${O}_{1}, {O}_{2}$ or both simultaneously. A node is only in ${O}_{2}$ if it is the center of an isolated star, that is, the rest of the network nodes are connected to it. By assuming that networks have a more complex structure, no nodes can touch exclusively ${O}_{2}$. However, a node can touch exclusively ${O}_{1}$. This occurs in cases where nodes have only one connection, or they are the non-common vertex of two triangles that share one or two vertices. Accordingly, leaf nodes are defined by: ${P}_{1,i}=1$, ${P}_{2,i}=0$ and ${P}_{3,i}=0$. Nodes that are not leaf nodes or vertices of a triangle are defined by: ${P}_{1,i}=1$, ${P}_{2,i}=1$ and ${P}_{3,i}=0$. When ${\rho }_{2}$ is close to one, the presence of leaf nodes is high. The lower this coefficient, the lower the number of nodes that have just one connection; this is characteristic of star graphs.

5 $${\rho }_{2}=1- \frac{\sum_{i}{P}_{2,i}(1- {P}_{3,i})}{\sum_{i}{P}_{1,i}(1- {P}_{3,i})}$$

#### Leaf-base strength, ${{{\rho}}}_{3}$

This ratio analyses if leaf nodes are connected to either hubs or low-degree nodes. This is the average number of times leaf nodes touch ${O}_{1}$(6). The value of ${O}_{1}$ for leaf nodes is equal to the degree of its neighbor. Thus, the higher the value of ${O}_{1}$, the higher the degree of the node to which they are connected. Large values of ${\rho {^{\prime}}}_{3}$ may signal the presence of hubs in the network.

6 $${\rho {^{\prime}}}_{3}= \frac{\sum_{i}{O}_{1,i} {P}_{1,i} (1- {P}_{2,i})(1- {P}_{3,i})}{\sum_{i}{P}_{1,i} (1- {P}_{2,i})(1- {P}_{3,i})}$$

This dimension might be scaled with the maximum value of node degree, $\text{max}({O}_{0,i})$, in the network (7). ${\rho }_{3}$ can be rewritten as (8). If ${\rho }_{3}$ tends to zero, leaf nodes are connected to low-degree nodes. They may be the end nodes of node strings.

7 $${\rho }_{3}=\frac{{\rho {^{\prime}}}_{3}}{\text{max}({O}_{0,i})}$$

8 $${\rho }_{3}= \frac{\sum_{i}{O}_{1,i} {P}_{1,i} (1- {P}_{2,i})(1- {P}_{3,i})}{\sum_{i}{P}_{1,i} (1- {P}_{2,i})(1- {P}_{3,i})} \frac{1}{\text{max}({O}_{0})}$$

### Hubs

#### Hub coefficient, ${{{\rho}}}_{4}$

This dimension studies whether there is a tendency to form hubs in the network or not. It measures the average number of times nodes touch ${O}_{2}$ (9). All nodes touch ${O}_{2}$ except for leaf nodes and nodes that are only in ${G}_{2}$(they are only vertices of triangles). The larger the number of connections of a node, the larger the value of ${O}_{2,i}$. Large values of ${\rho {^{\prime}}}_{4}$ therefore shows there is a tendency to make hubs in the network. Unlike ${\rho }_{3}$, the hub coefficient does not linearly correlate with node degree; ${O}_{2,i}$ is given by the binomial coefficient $\left(\genfrac{}{}{0pt}{}{n}{2}\right)$ where $n$ is the number of non-connected edges attached to node $i$ when the ${O}_{0,i}$ is greater than 2. If two networks have similar values of ${\rho }_{1}$, but different values of ${\rho {^{\prime}}}_{4}$, there is a higher tendency to make hubs in one network than in the other.

9 $${\rho {^{\prime}}}_{4}= \frac{\sum_{i}{O}_{2,i}}{\sum_{i}{P}_{2,i}}$$

To range between 0 and 1, ${\rho {^{\prime}}}_{4}$ can scale with the maximum value of ${O}_{2,i}$ in the network (10). ${\rho }_{4}$ can be rewritten as (11).

10 $${\rho }_{4}=\frac{{\rho {^{\prime}}}_{4}}{\text{max}({O}_{2,i})}$$

11 $${\rho }_{4}= \frac{\sum_{i}{O}_{2,i}}{\sum_{i}{P}_{2,i}} \frac{1}{\text{max}({O}_{2})}$$

#### Hub-connectivity coefficient, ${{{\rho}}}_{5}$

It analyzes if hubs tend to connect among them. This dimension is defined by the Spearman’s rank correlation between ${O}_{1}$ and ${O}_{2}$, (12) where $cov({rg}_{{O}_{1}},{rg}_{{O}_{2}})$ is the covariance of the rank variables of ${O}_{1}$ and ${O}_{2}$ and ${\sigma }_{{rg}_{{O}_{1}}}$,${\sigma }_{{rg}_{{O}_{2}}}$ are the standard deviation of both rank variables. This is one of the correlations proposed by Yaveroğlu et al.^24^. If ${\rho {^{\prime}}}_{5}$ tends to 1 means that nodes with high ${O}_{2}$ are also nodes with high values of ${O}_{1}$. The number of times a node touches ${O}_{1,i}$ increases with the degree of a node and its neighbors’ degree. However, the value of ${O}_{2,i}$ only depends on node degree; the higher the number of connections of a node, the higher the value of ${O}_{2,i}$. Consequently, nodes with a high value for ${O}_{1}$ and ${O}_{2}$ have a high node degree, they are hubs, and they are connected to other hubs. Therefore, a value close to 1 means that hubs tend to connect among them.

12 $${\rho {^{\prime}}}_{5}= \frac{cov({rg}_{{O}_{1}},{rg}_{{O}_{2}})}{{\sigma }_{{rg}_{{O}_{1}}}{\sigma }_{{rg}_{{O}_{2}}}}$$

This dimension is also scaled to range from 0 to 1 (13). ${\rho }_{5}$ can be rewritten as (14).

13 $${\rho }_{5}=\frac{{\rho {^{\prime}}}_{5}}{2}+ \frac{1}{2}$$

14 $${\rho }_{5}=\frac{1}{2} \frac{cov({rg}_{{O}_{1}},{rg}_{{O}_{2}})}{{\sigma }_{{rg}_{{O}_{1}}}{\sigma }_{{rg}_{{O}_{2}}}}+ \frac{1}{2}$$

### Strings

#### String coefficient, ${{{\rho}}}_{6}$

This coefficient measures the proportion of nodes in the network that are in the middle of a string. A string is formed by two end nodes (one or both nodes are linked to the rest of the network and there is no edge connecting them) and a set of intermediate nodes that are connected consecutively and have no links with the rest of the network. Consequently, a node is in the middle of a string if it has two connections, it touches $${O}_{2,i}$$ only once ($${U}_{2,i}=1)$$ and it is not a vertex of a triangle ($${U}_{3,i}=1$$). Therefore, $${\rho }_{6}$$ is the ratio between the number of nodes that are in the middle of a node string and the total number of nodes that touch $${O}_{2}$$ (15). Not all degree-two nodes touch $${O}_{2}$$ once (triangle vertices do not touch $${O}_{2}$$). In addition, not all nodes that touch $${O}_{2}$$ once are in the middle of a node string. A node might touch $${O}_{2}$$ only once if it is a shared vertex of a triangle ($${O}_{3,i}>0$$ and $${U}_{3,i}=0)$$, so the node is not part of a string.

15 $${\rho }_{6}=\frac{\sum_{i}{{U}_{2,i}U}_{3,i}}{\sum_{i}{P}_{2,i}}$$

16 $${U}_{2,i}=\left\{\begin{array}{c}1, {O}_{2,i}=1\\ 0, {O}_{2,i}\ne 1\end{array}\right.$$

17 $${U}_{3,i}=\left\{\begin{array}{c}1, {O}_{3,i}=0\\ 0, {O}_{3,i}\ne 0\end{array}\right.$$

#### Characteristic string length, ${{{\rho}}}_{7}$

This dimension is the average length of node strings (considering only middle nodes and disregarding the end nodes of the string) in the network as shown in (18), where $n$ is the number of node strings in the network.

18 $${\rho {^{\prime}}}_{7}=\frac{\sum_{i}{U}_{2,i}{U}_{3,i}}{n}$$

To enhance network comparison, ${\rho {^{\prime}}}_{7}$ is scaled as its inverse (19). If ${\rho }_{7}$ is equal to zero, it means that all node strings have two end nodes and only one middle node.

19 $${\rho }_{7}=1- \frac{n}{\sum_{i}{U}_{2,i}{U}_{3,i}}$$

### Triangles

#### Triangle rate, ${{{\rho}}}_{8}$

This coefficient studies whether there is a tendency to make triangles in the network or not. It measures the proportion of triangles (${G}_{2}$) in a network with respect to the total three-node graphlets (20). The number of ${G}_{2}$ in the network is equal to $\frac{\sum_{i}{O}_{3,i}}{3}$ and the number of ${G}_{1}$ is equal to $\sum_{i}{O}_{2,i}$. This ratio is similar to the global clustering coefficient. However, many works in the literature use the network average clustering coefficient to analyze network properties. The network average clustering coefficient weights more nodes with a low degree (as discussed in the Supplementary Information Sect. 2). Thus, it is not a correct measure to analyze network with a non-homogenous degree distribution. The average network clustering coefficient, therefore, differs from the value of ${\rho }_{8}$ which considers the whole topology of the network.

20 $${\rho }_{8}=\frac{\sum_{i}{O}_{3,i}}{3\sum_{i}{O}_{2,i}+\sum_{i}{O}_{3,i}}$$

#### Triangle concentration, ${{{\rho}}}_{9}$

This coefficient shows if triangles tend to be concentrated in networks. Triangles are concentrated when there are nodes that are vertices of two or more triangles. The dimension ${\rho }_{9}$ is complementary to the ratio between the number of nodes that are vertices of triangles and the number of triangles in the network (21). The higher the number of triangles that share some vertices the lower the value of ${\rho }_{9}$. If triangles have no shared vertices, the maximum value of ${O}_{3,i}$ is 1, and ${O}_{3,i}= {P}_{3,i}$. Therefore, the number of nodes that are in a triangle is three times the number of ${G}_{2}$ in the network (${3 G}_{2}= \sum_{i}{O}_{3,i}=\sum_{i}{P}_{3,i}).$ However, if triangles share vertices, $\sum_{i}{P}_{3,i}<{3 G}_{2}$. As ${\rho }_{9}$ converges to 0, the number of graphlets of type ${G}_{7}$, ${G}_{8}$, ${G}_{17}$, ${G}_{19}$, ${G}_{22}$, ${G}_{23}$, ${G}_{24}$, ${G}_{25}$, ${G}_{26}$, ${G}_{27}$, ${G}_{28}$ and ${G}_{29}$ (graphlets composed of triangles with shared vertices) converges to 0 too.

21 $${\rho }_{9}=1- \frac{\sum_{i}{P}_{3,i}}{\sum_{i}{O}_{3,i}}$$

#### Triangle pervasiveness, ${{{\rho}}}_{10}$

This dimension analyzes if triangles tend to cover the whole network or if they are concentrated around a few nodes. It measures the proportion of nodes in the network that are vertices of triangles (22). If a node is a vertex of a triangle, ${P}_{3,i}=1.$ As explained, in connected graphs, the number of nodes in a network is $\sum_{i}{P}_{0,i}$. This coefficient compliments ${\rho }_{8}$ and ${\rho }_{9}$, since it sheds light on whether triangles form a mesh that comprises most nodes in a network or not. A high value of ${\rho }_{8}$ might be a consequence of networks in which triangles are connected to hubs and low-degree nodes have a non-meshed structure or networks in which all nodes are connected by a triangle mesh. Therefore, ${\rho }_{10}$ allows for the discernment between those types of networks, this coefficient would have a low value in the first case, and it would be close to one in the second network.

22 $${\rho }_{10}= \frac{\sum_{i}{P}_{3,i}}{\sum_{i}{P}_{0,i}}$$

#### Triangle connectivity, ${{{\rho}}}_{11}$

It measures if triangles are isolated in the network or they are part of a highly meshed structure. A triangle is isolated if one or two of its vertices are not connected to the rest of the network. Consequently, those vertices have only two connections, they touch ${O}_{1,i}$ and ${O}_{3,i}$ and they do not touch ${O}_{2,i}$. Thus, ${\rho }_{11}$ is the ratio between the number of triangle vertices that are not connected to other nodes (${U}_{2,i}$ = 1) and the total number of nodes that are vertices of triangles ($\sum_{i}{P}_{3,i}$) (23). The lower the value of ${\rho }_{11}$, the lower the number of isolated triangles in the network.

23 $${\rho }_{11}= \frac{\sum_{i}{P}_{3,i }{U}_{2,i}}{\sum_{i}{P}_{3,i}}$$

#### Triangle degree, ${{{\rho}}}_{12}$

This dimension shows if triangles tend to be connected to hubs or to low-degree nodes. It is the average degree of triangle vertices (24). That is the mean value of ${O}_{0,i}$ for those nodes that are in a triangle (${P}_{3,i}=1$). High values of ${\rho }_{12}$ mean that triangles are connected to hubs. The lower the value of ${\rho {^{\prime}}}_{12}$, the lower the average node degree of triangle vertices.

24 $${\rho {^{\prime}}}_{12}= \frac{\sum_{i}{O}_{0,i} {P}_{3,i} }{\sum_{i}{P}_{3,i}}$$

To range between 0 and 1, ${\rho {^{\prime}}}_{12}$ is scaled with the maximum value of node degree (25). ${\rho {^{\prime}}}_{12}$ can be rewritten as (26).

25 $${\rho }_{12}=\frac{{\rho {^{\prime}}}_{12}}{\text{max}({O}_{0,i})}$$

26 $${\rho }_{12}= \frac{\sum_{i}{O}_{0,i} {P}_{3,i} }{\sum_{i}{P}_{3,i}} \frac{1}{\text{max}({O}_{0})}$$

A summary table for the dimensions of the GHuST framework is shown in Supplementary Information Table S1.

## Explaining the topology of real networks

To prove the accuracy of the proposed method, this section applies the 12-dimensional metric to a set of five real networks. It aims to prove if the information provided by $\rho$ is consistent with the global-topological statistics usually used to describe network structure. These five networks have different sizes and display completely different structures, as shown in Supplementary Information Fig. S1. The two social networks and the metabolic network are in the range of 1,000 to 1,500 nodes, and the two infrastructure networks are two and five times larger, respectively. However, the number of edges is much higher in the social networks; in the case of the Facebook network, the number of edges is twenty times larger than in the road networks. Differences in network size obscure the comparison among networks with global statistics. In some cases, as in distance-based metrics, it is not always possible to infer if there is a change in a variable because of network size or network structure.

The five real networks are modeled as non-directed and unweighted networks to apply the GHuST framework. This framework does not consider edge direction or edge weight. Although an extension to weighted and directed networks is not the scope of this paper, the GHuST framework may include potentially both properties. On the one hand, edge direction leads to a different graphlet decomposition as pointed by Aparício et al.^26^. They propose 39 non-bidirectional directed graphlets of 2, 3, and 4 nodes. Then, graphlet and orbit definitions differ between directed and non-directed networks and new GHuST dimensions would apply. Those new dimensions, therefore, incorporate edge direction. While this will increase the complexity of the GHuST framework, it will provide a sounder analysis of network structure in the case of directed graphs. For instance, the inclusion of edge direction in the analysis of power networks will explain the role of leaf nodes in the network. That is, if leaf nodes inject or withdrawn power in the network. Consequently, the explanation given by the leaf rate (${\rho }_{2}$), will be completed with the direction of network edges that might represent power flow through lines. By defining new GHuST dimensions, we will differ between radial networks in which leaf nodes withdrawn power in the network, demand nodes, or nodes that inject power, power plants.

On the other hand, the inclusion of edge weight in the analysis of graphlet decomposition was covered by Azari and Airoldi^27^. However, the expansion of the GHuST framework to weighted networks would not be as straightforward as in the case of edge direction. The 12 GHuST dimensions should be completed with a set of coefficients that weight the importance of the edge in the network. Regarding the example above, values for the leaf rate should vary between networks with small power plants (e.g., wind and solar farms) and bigger power plants (e.g., thermal power plants) connected to the network through a single line. However, as stated, the inclusion of edge weight requires additional research to be effectively included in the GHuST framework.

This paper highlights the simplicity of the GHuST framework, which has been coded in Matlab as well as the code to count graphlets and orbits. This claims result from the need to count 2-node and 3-node graphlets, that is four orbits (${O}_{0}$, ${O}_{1}$, ${O}_{2}$, ${O}_{3}$). Since the GHuST framework only analyzes graphlets of orders 3 and below, it can be calculated in affordable times. As Hočevar et Demšar show, an increase in the number of graphlet nodes triggers the computation time in all the analyzed methods to count graphlets covered in their study (FANMOD, GraphCrunch, and Orca) ^15,16,28^. For example, the time needed to count the 5-node graphlets is between 10 and 100 times higher than the time required to count the 4-node graphlets. The same increase is observed by Melckenbeeck et al. in their comparison when going from 5-node to 6-node graphlets^29^. Keeping the counting to order 3 and below allows for a manageable computational burden. The computational complexity of this counting is approximately of the order of $O\left(\left|V\right|{d}^{k-1}\right)$, where $V$ is the set of vertices, $d$ is the maximum degree and $k$ is the order of the graphlet counting, therefore yielding $O\left(\left|V\right|{d}^{2}\right)$ for our case. In addition, the calculation is easily parallelizable as expressed in published works^30^.

A thoughtful analysis of the graphlet distribution of each network has been carried out (see Supplementary Information Sect. 2). The results show that in our case, graphlet distribution is not an accurate tool to infer the topological properties of such complex networks, providing an incomplete description of the underlying network structure.

The proposed method overcomes the limitations of graphlet distributions to explain network topology by a 12-dimensional metric. To analyze results, Table 1 shows a set of global statistics used to analyze the five real networks, and Table 2 shows the value of the GHuST framework for those networks. (Values in Table 2 are not scaled, the reader is referred to Supplementary Information Sect. 2 for an in-depth analysis). The description provided by the GHuST framework of the topological structure of the networks is consistent with global statistics traditionally used in complex networks, overcoming their main drawbacks.

**Table 1:** Global topological properties of five real networks.

	$N$	$L$	$D$ (%)	$\langle k\rangle$	$\text{max}(k)$	Ass. coeff	$\langle l\rangle$	$d$	$\langle BC\rangle$	$\text{max}(BC)$	$\langle cc\rangle$
Road	2,642	3,303	0.02	2.5	5	− 0.187	35.35	99	4.52 × 10^4^	6.95 × 10^5^	0.016
Power-grid	4,941	6,594	0.03	2.7	19	0.004	18.98	46	4.44 × 10^4^	3.51 × 10^6^	0.080
Mail	1,133	5,451	0.43	9.6	71	0.078	7.21	8	1.47 × 10^3^	2.52 × 10^4^	0.220
Social	1,446	59,589	2.85	82.5	375	0.067	2.22	6	887	1.88 × 10^4^	0.323
Metabolic	1,039	4,741	0.44	9.13	638	− 0.251	2.47	6	766	2.46 × 10^5^	0.377

**Table 2:** Values of GHuST dimensions for a set of five real networks.

	${{\rho }^{^{\prime}}}_{1}$	${\rho }_{2}$	${\rho {^{\prime}}}_{3}$	${\rho {^{\prime}}}_{4}$	${\rho {^{\prime}}}_{5}$	${\rho }_{6}$	${\rho {^{\prime}}}_{7}$	${\rho }_{8}$	${\rho }_{9}$	${\rho }_{10}$	${\rho }_{11}$	${\rho {^{\prime}}}_{12}$
Road	0.25	0.04	1.85	2.18	0.72	0.57	1.55	0.01	0.03	0.05	0.01	3.42
Power grid	0.34	0.31	3.520	4.86	0.78	0.42	1.53	0.04	0.51	0.19	0.23	4.43
Email	3.81	0.51	16.25	85.97	0.96	0.08	1.04	0.06	0.95	0.74	0.06	12.34
Social	40.21	0.61	93.06	3,965.03	0.98	0.01	1.00	0.10	1.00	0.98	0.01	84.02
Metabolic	3.56	0.04	321.00	472.05	0.80	0.06	1.06	0.01	0.96	0.84	0.05	10.31

${\rho }_{i}$ is the dimension $i$ of the GHuST framework. Dimensions 1, 3, 4, 5, 7, and 12 are not scaled, this table shows the values for ${\rho {^{\prime}}}_{i}$.

### A panoramic view offered by local properties

The previous section has illustrated the application of the proposed metric as a tool for summarizing the main topological features of complex networks. This section aims at evaluating the performance of this technique using a large sample, 1,404 graphs, of real networks from different domains: Autonomous Systems, Enzymes, Facebook, Power Network, Retweet, Roads, and Web.

The autonomous-systems set stands for 733 daily instances of graphs of routers comprising the internet^31^. The enzymes, Facebook, retweet, roads, web, and some power-network graphs are obtained from an open-access network repository^32^. The enzyme dataset includes 476 samples (the analysis only considers graphs with more than 20 nodes). The Facebook set consists of 108 networks of friendship connections. The power-network graphs comprise the transmission (220 kV and 400 kV) power networks of fifteen European countries, and a set of power networks (7 graphs) obtained from the open-access repository (voltages levels are not specified)^32,33^. The retweet networks form a set of 32 graphs. The road set includes 16 instances. Finally, 17 networks are part of the web graphs.

Once we compute the 12-dimensional metric for each network, Principal Component Analysis (PCA) is used to validate the usefulness of the proposed statistic. In addition, to enabling visual inspection of our data, it can be used to verify if some dimensions of the 12-dimensional metric could be removed. PCA is a statistical technique that seeks to obtain a linear combination of the original variables in such a way that the maximum variance is explained. This allows us to obtain a low-dimensional representation of the data that captures most of the original information. As there are no null coefficients in the PCA loadings, it can be concluded that there are no redundant dimensions in the proposed metric. Furthermore, any network with unusual topological properties will be highlighted in our analysis, providing a tool for detecting outliers. Varimax rotation was applied to improve the understanding of PCA analysis. The varimax technique pursues obtaining principal components that are easier to interpret, by rotating the original principal components in such a way that each one is strongly correlated with as few of the original variables as possible. However, results obtained with varimax rotation did not improve the results shown in this section.

**Figure 2:**
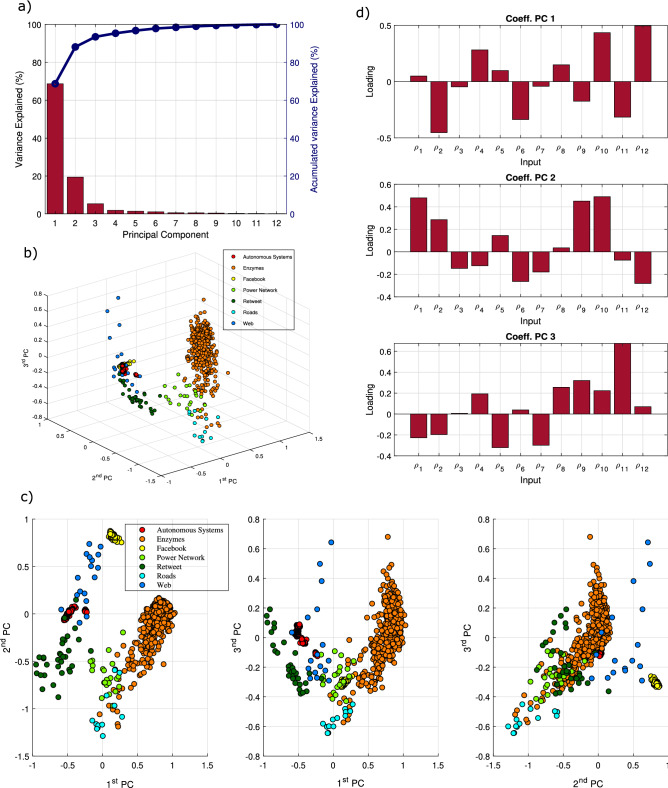
(**a**) Variance explained (%) and accumulated variance explained (%) by each of the principal components resulting from the PCA analysis to a set of 1,404 networks. As can be seen, the first 3 principal components summarize more than 90% of the variance of the original data. (**b**) Graphical representation of the 1,404 networks in the 3-d space defined by the three first principal components. In this representation, several clusters can be appreciated, corresponding to networks with similar topological structure. (**c**) 2-d projections of the 3-d representation of the 1,404 networks in the space defined by the three first principal components. (**d**) Loadings of $\rho$ for the three first principal components. As there are no null coefficients, it can be concluded that there are no irrelevant dimensions in the proposed 12 dimensional metric.

Figure 2a shows the proportion of variance explained by the principal components. By selecting the first three components, we are able to capture 93.4% of the variance of the original data, allowing us to obtain a low-dimensional view of the distribution of our data. The weights of the 12 dimensions of our metric for each component are shown in Fig. 2d, and they can be used to obtain an interpretation of each component. The first component (68.7% of variance), accounts for a positive contribution of ${\rho }_{4}$, ${\rho }_{10}$, ${\rho }_{12}$ and a negative contribution of ${\rho }_{2}$, ${\rho }_{6}$ and ${\rho }_{11}$. Therefore, the main topological differences among the networks analyzed lie on the proportion of leaf-nodes, presence of hubs and strings, as well as the triangle pervasiveness and connectivity coefficients and triangle degree. A similar interpretation can be obtained for the second component (19.4% of variance) and the third component (5.3% of variance) based on Fig. 2d.

By projecting the coordinates of our 12-dimensional data on the space spanned by the first 3 principal components, we can visualize the distribution of the metric for each network in this new axis system. As seen in Fig. 2b,c, networks from different processes tend to have similar topological properties, hence showing clear groupings in the principal-component space.

The autonomous-system and Facebook networks form two clearly bounded clusters in the three-first principal-component space. Despite being the category with more instances, all the autonomous-system instances are close to − 0.5 in the first component and to 0 in the second and third components. Since in the first principal component, ${\rho }_{i}$ have positive and negative loadings, we cannot state if those values close to zero are the consequence of low values of all components, or they are the consequence of the balance between positive and negative loadings. Tables include in Supplementary Information Sect. 4 show the range in which the 12 dimensions vary. The analysis of ranges for each type of network allows for the classification of graphs. In the case of Facebook graphs, most analyzed instances have values of ${\rho }_{3}$, ${\rho }_{4}$, ${\rho }_{6}$, ${\rho }_{7}$, ${\rho }_{8}$, ${\rho }_{11}$, ${\rho }_{12}$ that are close to zero and the values of ${\rho }_{1}$, ${\rho }_{5}$, ${\rho }_{9}$, ${\rho }_{10}$ are close to one. In Supplementary Information, the reader can find a detailed explanation of ${\rho }_{i}$ distribution for each type of network.

Regarding the two infrastructure networks, roads and power networks comprise two independent clusters. Although some road networks are close to some power grids in the space defined by the first and second principal components, they are clearly delimited in the other two projections of the three first principal components.

Both roads and power networks have low values for the second component, that is low values of ${\rho }_{1}$, ${\rho }_{9}$ and ${\rho }_{10}$. Accordingly, the number of connections in comparison with the minimum spanning tree is low, there is a low number of triangles in the network and they do not tend to share vertices. The instances of roads and power networks that have similar values for the second principal component have a similar number of edges per node. They are the power networks with the lowest number of lines per node with respect to other power networks and the roads with a higher number of lines per node in their category.

Unlike social networks, connections in infrastructure networks are cost-intensive and they are conditioned by topological, morphological, technical, economical, permitting, environmental, managerial, and political factors^34^. Consequently, the influence of all those factors may lead to different topological properties depending on regions. Furthermore, in the case of power networks, graphs may include different voltage levels or they may be the result of different model assumptions^35^. This uncertainty leads to a lack of consensus about some of the topological properties of power networks^36^.

**Figure 3:**
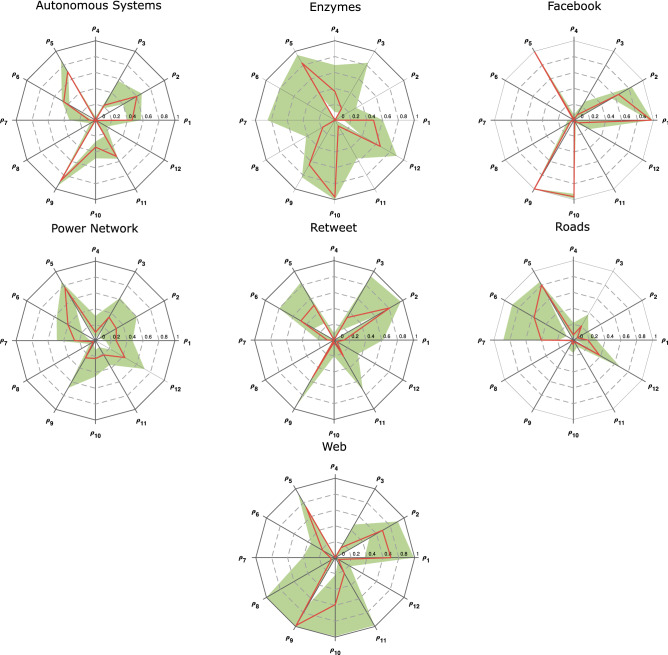
Range of variation of each metric dimension, ${\rho }_{i}$, (green area) and median value of each ${\rho }_{i}$ for the seven set of networks analyzed: autonomous systems, enzymes, Facebook, power networks, retweet, roads and webs. Networks with different structures exhibit a distinct distribution of the ${\rho }_{i}$ values, as highlighted by these charts.

The cluster with the most variation among its members belongs to the enzymes group. This shows that a network cannot be classified in the enzyme group as clearly as, for instance, Facebook networks. The green area that shows the range in Fig. 3 almost covers all the dodecagon. The topological properties of enzymes are clearly case dependent.

Finally, we can also see two clusters considering the web and retweet group. In the case of web networks, there is a large variation in the third component. It ranges from − 0.3 to 0.7. This variation is caused by the significant difference in ${\rho }_{11}$ (triangle-connectivity coefficient). Although the median of the analyzed instances has a low value, this coefficient ranges from 0 to 1. In the web case, we also see that although most instances have a triangle coefficient (${\rho }_{8}$) close to zero, there is an instance in which ${\rho }_{8}$ tends to 1 (the network is mainly formed by triangles). This coefficient is coherent with the network average clustering coefficient^32^. Accordingly, this framework also supports the quick detection of potential outliers.

**Figure 4:**
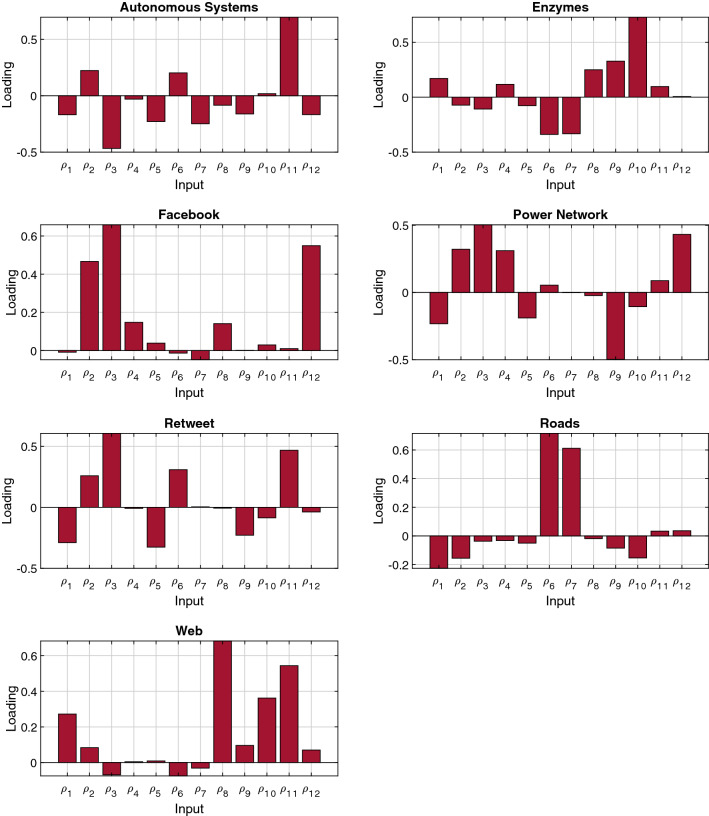
Contributions of each dimension of the GHuST framework to the first principal component obtained for each set of networks analyzed. Larger loadings are associated with a high variance in the original dataset, providing information about the topological differences between networks of the same set.

**Figure 5:**
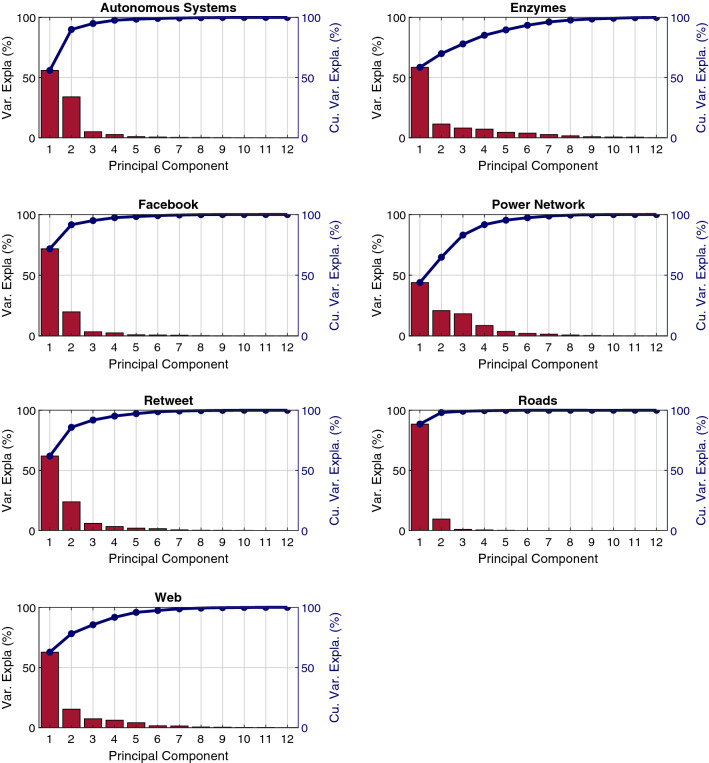
Variance explained and cumulative variance explained by each of the principal components of the resulting PCA applied independently to each type of network analyzed. As can be seen, in all cases using the first 3 principal components account for more than 90% of the variance of the original dataset.

PCA analysis can be used for each set of networks independently. Therefore, the dimensions with larger loadings for the first components are the ones that exhibit the most variance in each original set, hence those dimensions will provide information about the topological differences between networks of the same set. Dimensions that have similar values for all networks in the set will have a low contribution to the first components as they are characteristics of those networks. The explained variance for each principal component and the coefficient that shape the first component are shown in Figs. 4 and 5 respectively. A low dimensional representation of the projections of the metrics in the three first principal components for each set of networks can be seen in (Supplementary Information Sect. 3). In the case of the road networks, the first component explains 88.5% of the variance. This component is mainly defined by ${\rho }_{6}$ and ${\rho }_{7}$. Therefore, the difference among roads networks lies on the number of nodes that are part of a node string in the network and the length of those strings.

When analyzing power networks, we observe that the first component only explains 44% of the variation. Consequently, the number of coefficients to describe and to explain differences among power networks is larger. The first component is mainly described by ${\rho }_{1}$, ${\rho }_{2}$, ${\rho }_{3}$, ${\rho }_{4}$, ${\rho }_{9}$, ${\rho }_{12}$. It is necessary to include five principal components, to explain 95% of the variance of the data. This increases the number of metric dimensions required to have a deep understanding of power-network topology. In the case of Facebook, the first component explains 72% of the variance. Consequently, the main differences lie in the leaf coefficient, leaf-connection degree, and triangle degree.

Additionally, two more dimensionality reduction techniques have been implemented to compare the results of the PCA. Firstly, Independent Component Analysis (ICA), proposed by Hyvärinen et al.^37^, is employed to compare its results with those of the PCA. Unlike PCA, ICA tries to project the original data into a subspace where they are maximally independent. This technique is often used to uncover hidden structures in the original data. Secondly, a Self-Organizing Map (SOM) proposed by Kohonen^38^, is fitted, and its results are compared to the PCA low-dimensional representation. The SOM is a competitive learning algorithm that tries to find a low-dimensional representation of the data in such a way that the topological ordering properties of the original data are preserved. These two models are analyzed in Supplementary Information Sect. 5, where both models are compared with the PCA study carried out in this section. The results show a similar ordination of the networks in both models, validating the PCA study. Clusters obtained by these new two methods match those obtained with the principal components, highlighting the capabilities of the proposed method to explain network topology.

Results show the strengths of the proposed method to compare networks of different nature and to find the topological differences among same-nature networks.

## Conclusions

The analysis of network graphlets, a local-topological statistic, gives rise to a new description of the global topology of complex networks. This paper introduces an innovative method that analyzes the interaction among graphlets to explain and characterize network topology. This method is based on 2- and 3-node graphlets (three graphlets and four orbits) that are easily derived from the adjacency matrix. Therefore, it overcomes the limitation of counting high degree graphlets that might be cost-intensive for large networks.

The application of the novel framework to five real networks shows that the proposed method is consistent with the global statistics traditionally used to characterize network structure. Furthermore, it overcomes two of their main drawbacks: the use of metrics based on average values and the application of metrics that do not scale linearly with network size. Accordingly, the comparison among networks of different sizes does not require any analysis of metric scalability.

The proposed method has been also validated with a large sample study of networks that arise in different fields. Results prove that the information provided by this novel metric can be used to identify the underlying topological features of the networks and even to provide us with a visual tool to distinguish networks with different properties.

Consequently, this method might explain the evolution in both local and global properties of networks in which growth affects the whole structure. It can also be used to compare networks where network growth does not necessarily imply a change in local properties. This is common in infrastructure networks.

Finally, this work sets up a systematic analysis consisting of a 12-dimensional metric, to explain the properties of the network structure. Moreover, the proposed method allows for the translation of topological properties into other scientific dimensional languages. This is possible because global properties are explained from local structures that are easily interpretable.

## Supplementary information

Supplementary Information.
