# Supplementary material for: Exploiting graphlet decomposition to explain the structure of complex networks: the GHuST framework
Source: Sci Rep. 2020 Jul 30;10:12884. doi: 10.1038/s41598-020-69795-1 (PMC7393148; doi:10.1038/s41598-020-69795-1)
Supplement: Supplementary file 1 — Supplementary Information. [file 41598_2020_69795_MOESM1_ESM.docx]

**Supplementary information for “*Exploiting graphlet decomposition to explain the structure of complex networks: the GHuST framework”***

Rafael Espejo^1^, Guillermo Mestre^1^, Fernando Postigo^1^, Sara Lumbreras^1^, Andres Ramos^1^, Tao Huang^2^, Ettore Bompard^2^

1. *Universidad Pontificia Comillas. ICAI. Instituto de Investigación Tecnológica. Madrid, Spain*
2. *Politecnico di Torino, Dipartmento Energia, Torino, Italy*

## Orbits calculation from the Adjacency matrix

The twelve dimensions of the GHuST framework are obtained from the decomposition of networks in 2-node and 3-node graphlets, comprising three graphlets and four orbits. The adjacency matrix succinctly reveals the number of times a node touches those orbits. In non-directed networks, the adjacency matrix is symmetric and the sum of the elements in the $i$th row (or $i$th column) is, therefore, the degree of a node or $O_{0,i}$ (SI-1).

| $O_{0,i}= \sum_{j} {Adj}_{i,j}, \forall j$ | (SI-1) |
| --- | --- |

The number of times a node $i$ touches $O_{1}$ is equal to the number of nodes $j$ that are connected to node $i$ by a two-edge path (through node $k$) (SI-2). If a node $j$ can be reached from node $i$ through one or two edges simultaneously, nodes $i$ and $j$ are vertices of a triangle and they touch $O_{3}$. Alternatively, the non-zero elements of ${Adj}^{2}$ show the number of two-edge paths that connect two nodes. However, this matrix does not consider if those nodes are vertices of a triangle or not.

| $O_{1,i}= \sum_{j} \sum_{k} \left( {Adj}_{i,j}{Adj}_{j,k} \right)\left( 1-{Adj}_{i,k} \right), \forall j,k \neq i$ | (SI-2) |
| --- | --- |

$O_{2,i}$ is the binomial coefficient $\binom{n}{2}$ where $n$ is the number of edges attached to a node that is not connected among them attached to node $i$.$O_{2,i}$ can also be obtained from (SI-3).

| $O_{2,i}= \sum_{j} \sum_{k} \left( {Adj}_{i,j}{Adj}_{i,k} \right)\left( 1-{Adj}_{j,k} \right), \forall j,k \neq i$ | (SI-3) |
| --- | --- |

As an extension of $O_{1}$, a node $i$ touches $O_{3}$ when it is the vertex of a triangle (SI-4). In this case, the number of times node $i$ is a vertex of a triangle is also equal to $\frac{1}{2}{Adj}_{i,i}^{3}$.

| $O_{3,i}= \sum_{j} \sum_{k} \left( {Adj}_{i,j}{Adj}_{i,k} \right)\left( {Adj}_{i,k} \right), \forall j,k \neq i$ | (SI-4) |
| --- | --- |

A summary table for the dimensions of the GHuST framework is shown in SI-Table 1.

SI-Table 1.
Name, definition, and values of GHuST dimensions.

| Name: | Definition: | Values: |
| --- | --- | --- |
| Line-surplus coefficient | $\rho_{1}=1- \frac{2 \sum_{i} P_{0,i}}{\sum_{i} O_{0,i}}$ | $\rho_{1}\to1$: highly meshed structure  $\rho_{1}\to0$: no meshed structure |
| Leaf rate | $\rho_{2}=1- \frac{\sum_{i} P_{2,i}(1- P_{3,i})}{\sum_{i} P_{1,i}(1- P_{3,i})}$ | $\rho_{2}\to1$: large presence of leaf nodes  $\rho_{2}\to0$: low presence of leaf nodes |
| Leaf-base strength | $\rho_{3}= \frac{\sum_{i} O_{1,i} P_{1,i} (1- P_{2,i})(1- P_{3,i})}{\sum_{i} P_{1,i} (1- P_{2,i})(1- P_{3,i})} \frac{1}{max(O_{0})}$ | $\rho_{3}\to1$: leaf nodes connected to high-degree nodes  $\rho_{3}\to0$: leaf nodes connected to low-degree nodes |
| Hub coefficient | $\rho_{4}= \frac{\sum_{i} O_{2,i}}{\sum_{i} P_{2,i}} \frac{1}{max(O_{2})}$ | $\rho_{4}\to1$: presence of hub nodes  $\rho_{4}\to0$: no presence of hub nodes |
| Hub-connectivity | $\rho_{5}=\frac{1}{2} \frac{cov({rg}_{O_{1}},{rg}_{O_{2}})}{\sigma_{{rg}_{O_{1}}}\sigma_{{rg}_{O_{2}}}}+ \frac{1}{2}$ | $\rho_{5}\to1$: hubs tend to connect to other hubs  $\rho_{5}\to0$: hubs do not tend to connect to other hubs |
| String coefficient | $\rho_{6}=\frac{\sum_{i} {U_{2,i}U}_{3,i}}{\sum_{i} P_{2,i}}$ | $\rho_{6}\to1$: high presence of strings  $\rho_{6}\to0$: low presence of strings |
| Characteristic string length | $\rho_{7}=1- \frac{n}{\sum_{i} U_{2,i}U_{3,i}}$ | $\rho_{7}\to1$: long strings  $\rho_{7}\to0$: short strings |
| Triangle rate | $\rho_{8}=\frac{\sum_{i} O_{3,i}}{3 \sum_{i} O_{2,i}+\sum_{i} O_{3,i}}$ | $\rho_{8}\to1$: high presence of triangles  $\rho_{8}\to0$: low presence of triangles |
| Triangle  concentration | $\rho_{9}= 1-\frac{\sum_{i} P_{3,i}}{\sum_{i} O_{3,i}}$ | $\rho_{9}\to1$: triangles tend to share vertices  $\rho_{9}\to0$: triangles do not tend to share vertices |
| Triangle  pervasiveness | $\rho_{10}= \frac{\sum_{i} P_{3,i}}{\sum_{i} P_{0,i}}$ | $\rho_{10}\to1$: most nodes are part of a triangle  $\rho_{10}\to0$: most nodes are not part of a triangle |
| Triangle  connectivity | $\rho_{11}= \frac{\sum_{i} P_{3,i}U_{2,i}}{\sum_{i} P_{3,i}}$ | $\rho_{11}\to1$: triangle vertices tend to be unconnected to the rest of network nodes  $\rho_{11}\to0$: triangle vertices tend to be connected to the network |
| Triangle degree | $\rho_{12}= \frac{\sum_{i} O_{0,i} P_{3,i}}{\sum_{i} P_{3,i}} \frac{1}{max(O_{0})}$ | $\rho_{12}\to1$: triangle vertices are high-degree nodes  $\rho_{12}\to0$: triangle vertices are low-degree nodes |

|  |  |
| --- | --- |
|  |  |
|  | |

SI-Fig. 1. **Graph representation of five real networks: the Minnesota road network (a), a power grid that represents the Western States Power Grid of the United States (b), the email interchanges network among members of a Spanish university (c), an extract of Facebook (d), and a network that stands for the metabolic reaction of the E.coli bacteria (e). Node color represents node degree for each network.**

## Graph representation of five real networks

This section applies the twelve-dimensional metric to a set of five real networks. This set includes: two infrastructure networks the Minnesota road network and a power grid that represents the Western States Power Grid of the United States^1,2^, two social networks: an extract of Facebook and the email interchanges among members of a Spanish university^2,3^, and a network that represents the metabolic reaction of the E.coli bacteria^4^. A graph representation for the five networks is shown in SI-Fig. 1. For this analysis, all networks are modeled as unweighted and undirected graphs.

### Graphlets a matter of interaction

Scalability is one of the problems when using graphlets to describe network topology. The number of graphlets that a node touches depends on its degree and its neighbors’ degree, but it also depends on the entire structure of the network. Subsequently, two networks with the same size (same number of nodes and edges) may have a different number of total graphlets. To make a comparison among networks regarding graphlets, we scale the frequency of a graphlet concerning the frequency of all same-size graphlets, as shown in SI-Fig. 2.

Considering 3-node graphlets, the distribution shows that the percentage of triangles $G_{2}$ looks extremely low in the five networks; in the metabolic and road network, the percentage of triangles is under 1.5%. Only in the network representing Facebook friendships does it reach 10%. However, a null model is necessary to compare results. Unless a network is formed exclusively by triangles, that is a network in which all nodes are connected among them, the frequency of $G_{1}$ in the network is not zero. Therefore, the value of $G_{2}$ has an upper bound.

| **Graphlet distribution: 3-node and 4-node graphlets** |
| --- |
|  |
| **Graphlet distribution: 5-node graphlets** |
|  |

**SI-Fig. 2. Distribution of 3- to 5-node graphlet (frequency of a graphlet concerning the frequency of all same-size graphlets) for five real networks**.

About 4- and 5-node graphlets, the frequency distribution shows that a few frequencies prevail over the rest. In the case of 4-node graphlets, $G_{5}$ to $G_{8}$ account for 9% of the power-grid and 2.5% of the road-network distribution. Similarly, in the metabolic network and in the road networks, $G_{12}$ to $G_{29}$ account for less than 7.4% of 5-node graphlets. The presence of more connected graphlets ($G_{12}$ to $G_{29}$) is only relevant to Facebook, where they represent 38% of 5-node graphlets.

Two graphlets dominate the metabolic network: $G_{4}$ (92% of 4-node graphlets) and $G_{11}$ (87% of 5-node graphlets). That distribution of frequencies contrasts with the other networks in which predominant frequencies are $G_{3}$ (4-node graphlets) and $G_{9}$ and $G_{10}$ (5-node graphlets). The number of times a node is in $G_{4}$ and $G_{11}$ is the binomial coefficient $\binom{n}{k}$ where $n$ is the number of non-connected edges attached to node $i$ and $k$ is three or four respectively. Therefore, those frequencies rapidly increase with the presence of hubs. The largest value of node degree in the metabolic network is 638 and the average node degree is 9.13, indicating a network with a few hubs connected to low-degree nodes. The predominance of those frequencies makes it impossible to infer a sound description of the metabolic network topology based on graphlet distribution will be limited to relatively low degrees.

In infrastructure networks, connections are cost-intensive and highly connected subgraphs are not as frequent as in social networks. If we compare the two infrastructure networks, we see that there is a lower tendency to make triangles in the road network (3-node graphlets). However, the number of triangles in the power grid is not necessarily larger than in the road network, since the total number of graphlets depends on network structure. In the power grid, the value of $G_{4}$ is twice larger than the road network. There are nodes with a higher degree than in the road network. Indeed, the global statistics show that the maximum degree is four times higher in the power-grid case. When analyzing 5-node graphlets, $G_{9}$and $G_{10}$ explain 95% and 75% of power-grid and road-network graphlet distribution, respectively. As in the prior case, the main conclusion is that the average node degree is higher in the power grid and local structures tend to be more connected than in the road networks (since highly connected graphlets have a slightly higher frequency). However, this information is not enough to characterize network topology accurately, and it might be misleading.

In the case of Facebook, it is not possible to infer if the values of $G_{9}$ and $G_{10}$ are because of the presence of hubs or not. This also requires a more in-depth analysis with a correct null model. When comparing the two social networks, the email network looks to have a less connected structure, since the frequency of highly connected graphlets in the email network is much lower than in the Facebook network. If we compare the mean absolute error ($MAE= \sum_{i} \frac{\left| G_{i,a-}G_{i,b} \right|}{n}$), the most similar networks in terms of graphlets frequencies are the power grid and the email network ($MAE \text{= 0.008}$). The $MAE$ between Facebook and email network is 0.021. However, when analyzing the global topological statistics (see Table 1), we see that the power grid and the email networks display entirely different structures.

Based on prior results, the use of graphlet distribution cannot infer the topological characteristics of complex networks. Other statistics should complete that topological analysis.

The analysis of graphlet distribution shows that in some networks, as in the case of infrastructure networks, only a few graphlets characterize network structure, so that calculating higher orders does not bring much additional information. Our method only uses $G_{0}$, $G_{1}$ and $G_{2}$. This reduces the complexity of measuring 30 graphlets and 72 orbits.

### Applying the GHuST framework

The GHust framework is applied to the five real networks to explaint their topological structure. Values for the twelve dimensions are shown in Table 2. Values shown in Table 2 are not scaled and they do not ncesesarily range between 0 and 1, SI-Fig. 3 shows the scaled values of GHuST dimensions.

### Global connectivity

The first dimension, ${\rho'}_{1}$, relates the number of nodes and edges. This dimension scales linearly with network size. This is a strength concerning other metrics such as edge density. While the number of edges to have a complete graph increases with $\Delta N(\Delta N-1)N_{0}$, where $\Delta N$ is the increase in nodes and $N_{0}$ the first set of nodes, the minimum number of lines to have a connected graph increases with $\Delta N$. In the five real networks used, there is no discrepancy in the order provided by edge density, $D$, and ${\rho'}_{1}$. However, there would have been discrepancies in the comparison of the following two networks: a network with 1,000 nodes and 2,000 edges and another network with 1,100 nodes and 2,200 nodes. The number of edges per node is the same in both networks. They have twice the number of edges needed by the minimum spanning tree, and there is no variation in ${\rho'}_{1}$. However, the edge density of the second network is lower than the edge density of the first (0.20% and 0.18% respectively). Therefore, results provided by ${\rho'}_{1}$ give a better understanding of the relation between the number of nodes and edges.

| 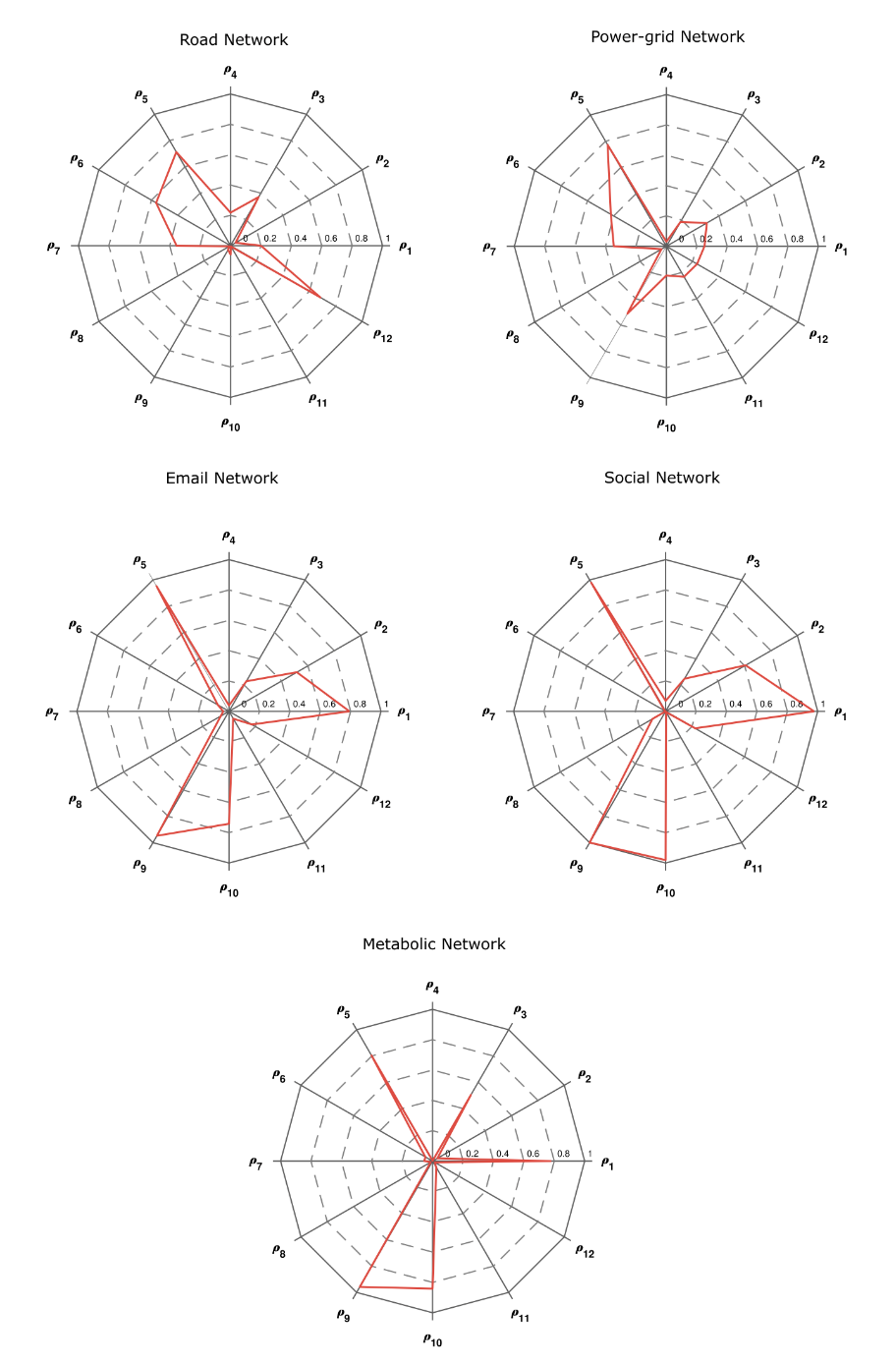 |
| --- |

**SI-Fig 3. Graphical representation of the GHuST framework for a set of five real networks**

For the infrastructure networks, ${\rho'}_{2}$ is lower in the power grid than in the road network. Indeed, the number of leaf nodes is 3.67% in the road network and 24.81% in the power grid. Therefore, we can infer that the power grid has nodes with a higher degree than the road network since the number of nodes per line and the percentage of nodes with only one connection is higher. The global statistic, maximum value of node degree, confirms that hypothesis.

In the case of the social networks, ${\rho'}_{2}$ is lower than in the other networks (0.607 in the Facebook network and 0.515 in the email network). However, the percentages of nodes with just one connection are 1.17% and 13.23%. The dimensions $\rho_{8}$ and $\rho_{9}$ explain this inconsistency. Both social networks have a significant presence of triangles concerning other networks (friends of friends tend to be friends themselves). Indeed, only 1.93% of Facebook nodes are not part of a triangle and 25.86% of nodes in the email network. Therefore, ${\rho'}_{2}$ only applies to those nodes that are not vertices of triangles. Accordingly, most nodes that are not vertices of a triangle are nodes with one connection. Similarly, in the metabolic network, 15% of nodes are not vertices of triangles, and the number of nodes with only one connection is scarce (0.5% of total nodes). Consequently, the value of ${\rho'}_{2}$ is 0.036 in the metabolic network.

The value of ${\rho'}_{3}$ shows that in the power grid the neighbors of nodes with one connection have a higher node degree than in the road network. Therefore, we infer that the road network has a more homogenous mesh, and the range of node-degree distribution is smaller than in the power grid which tends to create hubs, as shown in SI-Fig 1. The presence of hubs is also a characteristic of the metabolic network, where we see that ${\rho'}_{3}$ is 321. This value is considerably larger than the Facebook network with a greater number of edges per node. This leads to the existence of a small number of hubs that concentrate most connections. The ratio between maximum node degree and average node degree is 70, a huge value in comparison with the other four networks.

### Hubs

The tendency of a network to make hubs is supported by ${\rho'}_{4}$. As in the case of ${\rho'}_{3}$, the value of ${\rho'}_{4}$ is 5.5 times larger in the metabolic network than in the email network (both networks have a similar number of nodes and edges). The maximum degree is 638 in the metabolic and 71 in the email network. Both networks have a similar number of edges per node (${\rho'}_{1}$), the percentage of nodes with only one connection is lower in the metabolic network (${\rho'}_{2}$), and ${\rho'}_{3}$ is extremely large, so we may confirm the prior hypothesis that the high value of ${\rho'}_{4}$is the consequence of a few nodes with a high node degree. Accordingly, the third quantile of the degree distribution is 8 in the metabolic network and 13 in the email network. Therefore, although the hubs in the metabolic network have more connections on average, the number of nodes with a high degree is higher in the email network. In the case of the Facebook network, $\rho_{4}$ is slightly larger than in the metabolic and in the mail network. We infer that in Facebook, the difference between high-degree nodes and low-degree nodes is not so big as in the other two networks.

In the case of the two infrastructure networks, the higher the value of $\rho_{4}$ in the power grid reinforces our first insight about their network topological properties. Furthermore, ${\rho'}_{4}$ shows that in the road network the mean value of $O_{2}$ is closer to the maximum value. That means that the road network has a more homogeneous mesh than the power grid where there should be a few nodes with large values of $O_{2,i}.$

Finally, $\rho_{5}$, shows if hubs tend to connect to other hubs. That correlation is clear in the social networks. If we choose the 50 nodes with the highest degree in the Facebook network, we see those nodes have connections to 50% (average value) of those nodes. However, in the infrastructure networks and in the metabolic network we cannot state whether hubs tend to connect among them or not, ${\rho'}_{5}$ are around 0.75 in the range [0,1]. The values of ${\rho'}_{5}$diverge from the network assortativity coefficient, which has values close to zero (as shown in Table 1). Therefore, based on the network assortativity coefficient, nodes tend to connect high-degree nodes and low-degree nodes indifferently. The network assortativity coefficient measures if the degree of a node is correlated with its neighbors’ degree. A positive correlation means that high degree nodes have connections with other hubs. Furthermore, low degree nodes are connected to nodes with a low number of connections. By contrast, in a network with a negative correlation, low degree nodes are only connected to high degree nodes. Moreover, hubs are not connected among them. In large networks, this coefficient might be misleading. We cannot state if hubs tend to connect to other hubs considering the network assortativity coefficient since it is conditioned to the way in which low degree nodes are also connected. Because of network size, hubs might be connected to other hubs and low-degree nodes at the same time. Therefore, the network assortativity coefficient would be close to zero (there is not a linear correlation between node degree and its neighbors’ degree) and we will not obtain accurate information about the connection of hubs among them. The dimension ${\rho'}_{5}$ overcomes this limitation.

### Strings

Based on ${\rho'}_{5}$, we may think that in networks in which hubs connect among them, distances will be smaller. Accordingly, the characteristic path length of the Facebook network is 2, and the diameter is 6. Here, it is difficult to compare changes in the characteristic path length and the diameter since it does not scale linearly with network size. We see that the diameter is lower in networks with hubs (social and metabolic) than in the infrastructure networks. To reinforce this analysis, $\rho_{6}$ shows that in the two infrastructure networks there are many nodes that are part of strings. The presence of those strings on Facebook is almost zero, and the length of those strings, ${\rho'}_{7}$ is 1. Similarly, the closer the value of $\rho_{7}$ to 0, the shorter the node string. In the email network and in the metabolic network, there are a few more strings, but their length is also close to 0. However, in the infrastructure networks, there is a higher presence of strings. In the case of the road network, the average length of node strings is similar to the case of the power-grid network.

### Triangles

The dimensions $\rho_{8}$,$\rho_{9}$, $\rho_{10}$, $\rho_{11}$, $\rho_{12}$ supply detailed information about network clustering. As previously mentioned, the network average clustering coefficient, $\left\langle cc \right\rangle$, places more weight on low-degree nodes. In the case of the metabolic network, $\left\langle cc \right\rangle$ is 0.377. That might lead to the conclusion that nearly 40% of each node’s neighbors form a complete connected subgraph. However, this contrasts with $\rho_{8}$ that shows the metabolic network as the one with the lowest numbers of triangles. As shown in the graphlet distribution, less than 1.4% of three-connected nodes are triangles. The high value of $\left\langle cc \right\rangle$ concerning $\rho_{8}$shows that triangles in the metabolic network are connections of low-degree nodes. This is something that can be easily checked with $\rho_{10}$. In the metabolic network, the average degree of triangle vertices is 10.3, this value is close to the average node degree and far from the maximum degree in the network, 638. The number of edges needed by a node whose degree is 638 to have a value of local clustering coefficient equal to 1 is 215,644. Furthermore, $\rho_{9}$ shows that 96% of triangles share vertices, which reinforces the idea of low-degree nodes whose neighbors tend to form clusters. Those three dimensions explain network clustering, and they improve the information provided by the traditionally used network average clustering coefficient $\left\langle cc \right\rangle$.

The road network has a similar value of $\rho_{8}$. However, we see that more triangles do not share vertices; the average vertex degree is 3.41 and based on the first metric dimensions, we can conclude that the total number of triangles in the road network is lower (159 and 1,998 respectively). The total number of $G_{1}$ and $G_{2}$ in the road network is lower and therefore $\rho_{8}$ has similar values. To support this, we see that in the road network only 5% of nodes are vertices of a triangle, $\rho_{9}$. However, in the metabolic network, 84% of nodes are part of at least one triangle. Comparing the two infrastructure networks, the power grid has a higher number of triangles (651) and $\rho_{8}$ is larger. Unlike the road network, 26% of triangle vertices are not connected to the rest of the network. However, since in the power grid the maximum node degree is much higher than in the road network, the value of $\rho_{12}$ is higher in the road network. In the power grid, 50% of triangles share one of their vertices, there are more lines per node than in the road network ($\rho_{1}$) and more isolated nodes ($\rho_{2}$), this might lead to the conclusion that in the power grid there are more triangle vertices that are not connected to other nodes, (that is whose node degree is two). In the power grid, 20% of triangle vertices have degree equals to 2, in the road network that percentage is 0.6%. This is something that we see in $\rho_{11}$, 23% of nodes that are vertices of triangles have no more connections in the power grid.

Regarding the two social networks, both have a large number of triangles. In the case of Facebook, 10% of 3-node graphlets are triangles; this is a high value considering the presence of hubs, ${\rho'}_{4}$, which increases the number of total 3-node graphlets. Indeed, 98% of Facebook nodes are part of a triangle, as shown in $\rho_{10}$. Furthermore, almost all triangles share their vertices since $\rho_{9}$ is close 0. In the case of the email network, the presence of triangles in the network is 6%; this value is high in comparison with the network average clustering coefficient of another email network^5^. Only 5% of triangle vertices in the email network are not part of two or more triangles. Finally, if we compare the email network with the metabolic network, we can observe that in both networks ${\rho'}_{12}$ is similar. In the metabolic network, it looks like triangles are not part of hubs, since ${\rho_{12}}^{'}$ is much lower than the maximum node degree (low value of $\rho_{12}$).

## Loadings of the first 3 principal components for each set of networks

**
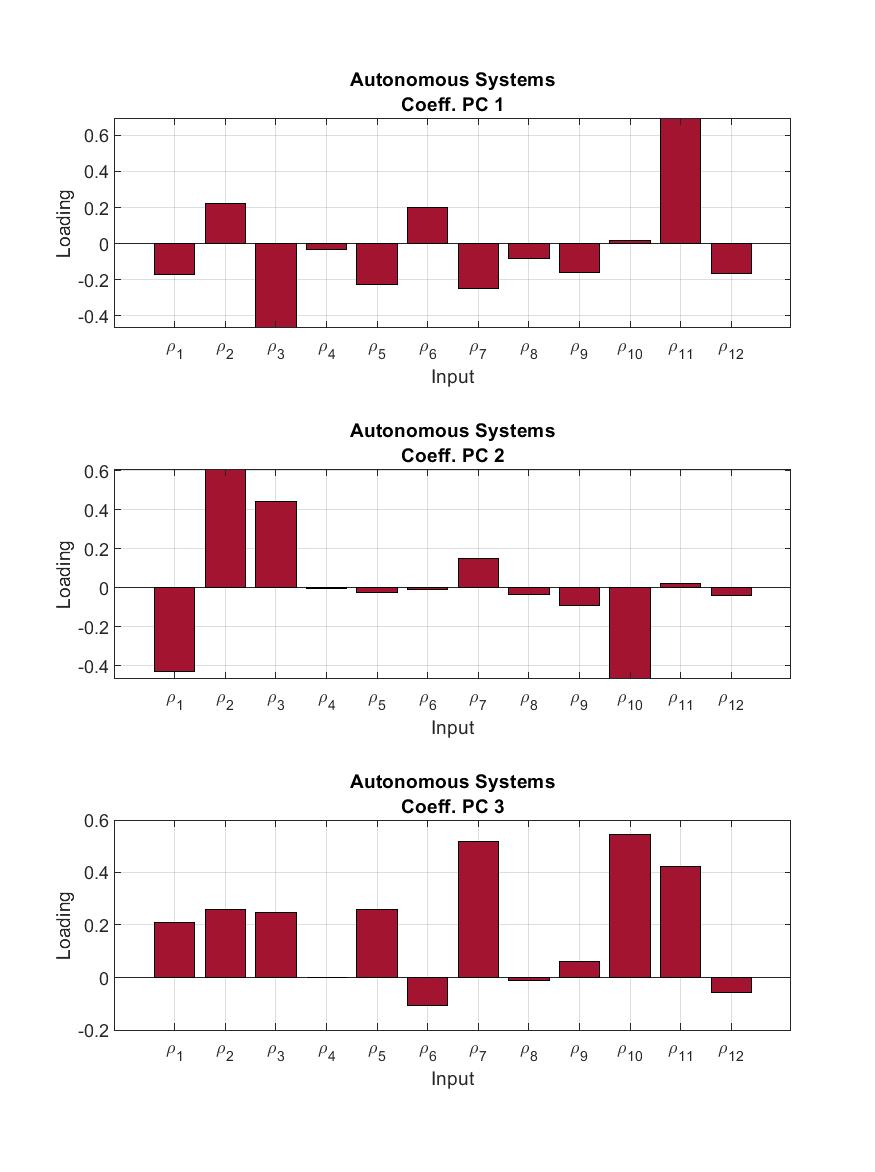
**

SI-Fig. 4. **Contributions of each component** $\boldsymbol{\rho}_{\boldsymbol{i}}$ **to the first 3 principal components obtained for the “Autonomous Systems” set of networks analyzed.**


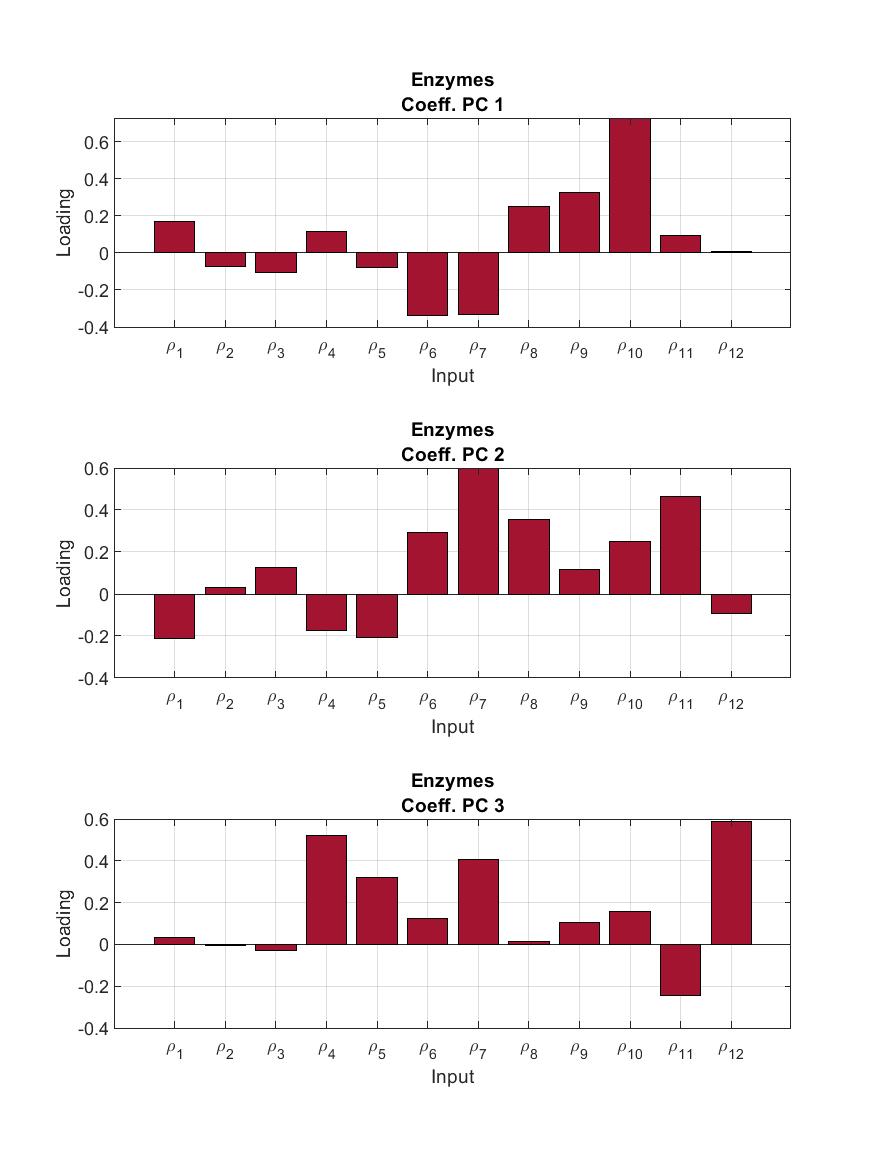


SI-Fig. 5. **Contributions of each component** $\boldsymbol{\rho}_{\boldsymbol{i}}$ **to the first 3 principal components obtained for the “Enzymes” set of networks analyzed.**

**
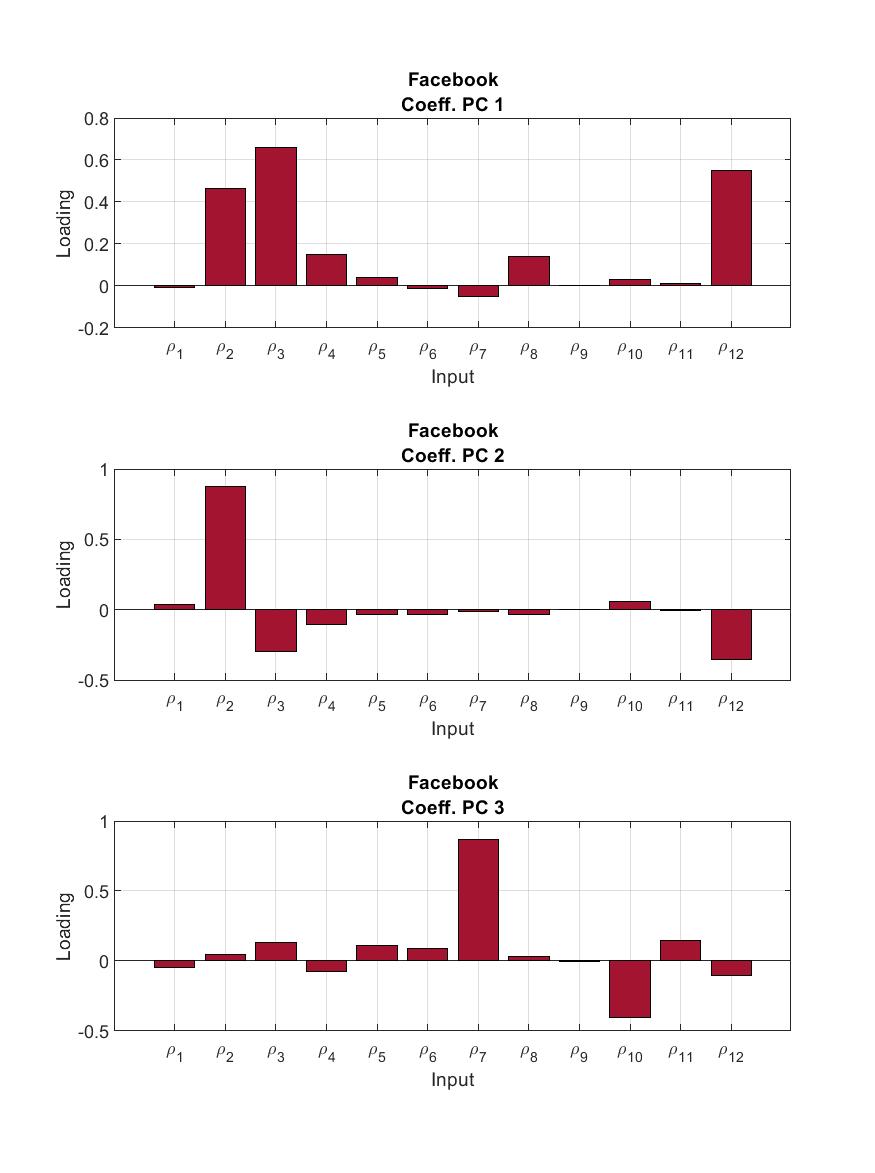
**

SI-Fig. 6. **Contributions of each component** $\boldsymbol{\rho}_{\boldsymbol{i}}$ **to the first 3 principal components obtained for the “Facebook” set of networks analyzed.**


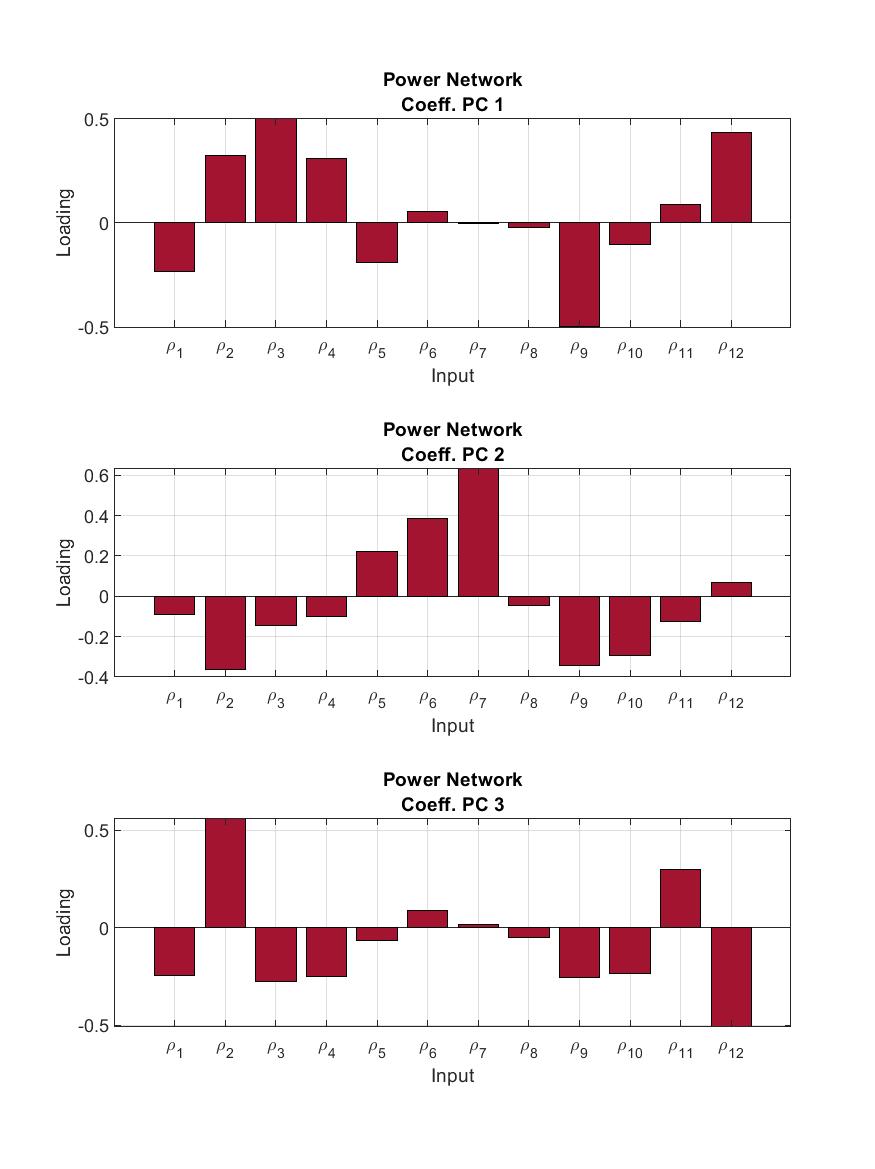


SI- Fig. 7. **Contributions of each component** $\boldsymbol{\rho}_{\boldsymbol{i}}$ **to the first 3 principal components obtained for the “Power Network” set of networks analyzed.**


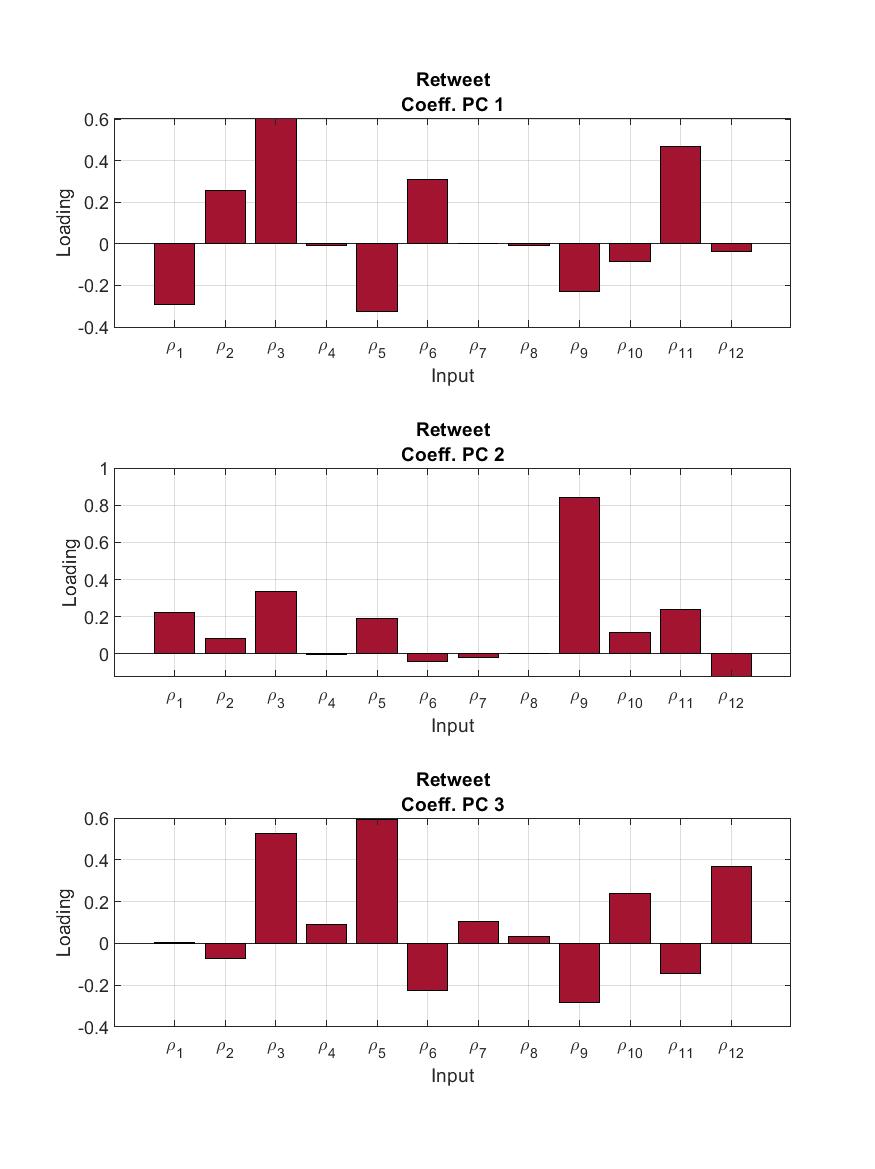


SI-Fig. 8. **Contributions of each component** $\boldsymbol{\rho}_{\boldsymbol{i}}$ **to the first 3 principal components obtained for the “Retweet” set of networks analyzed.**


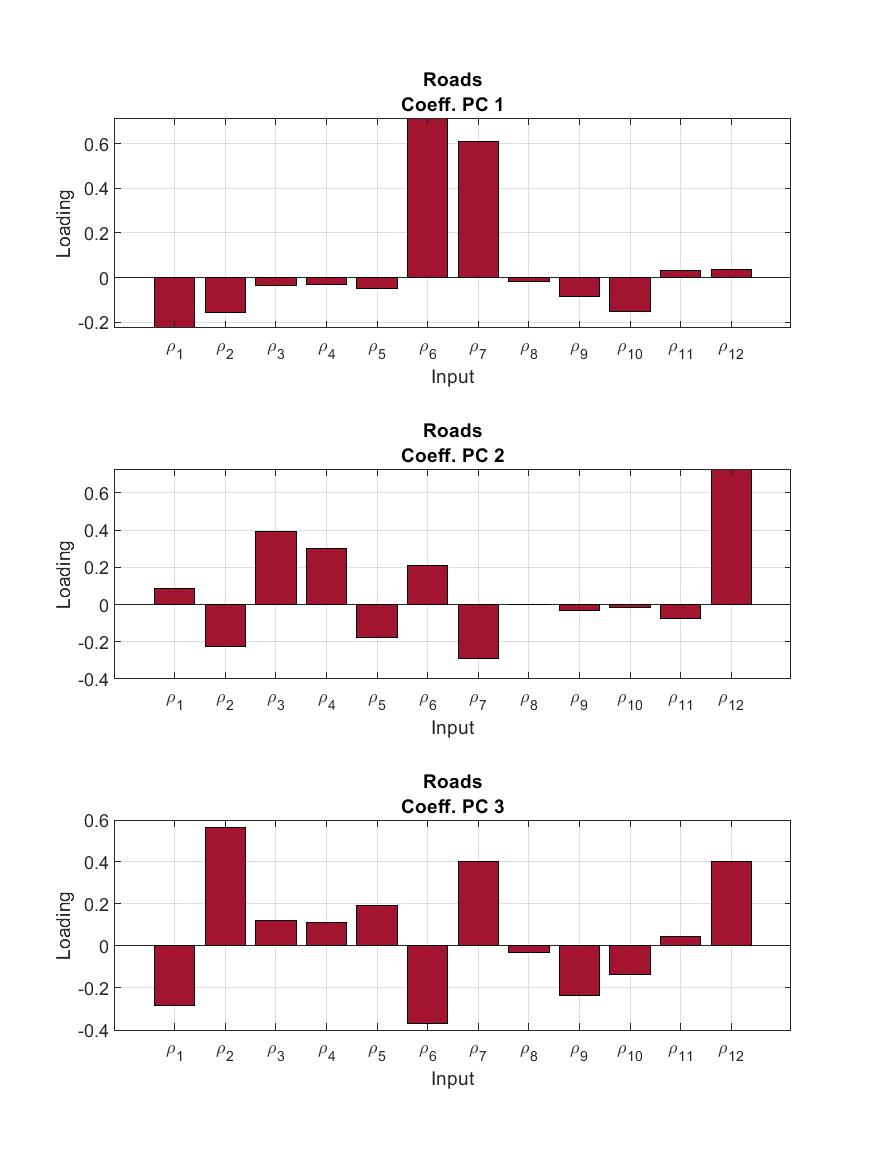


SI-Fig. 9. **Contributions of each component** $\boldsymbol{\rho}_{\boldsymbol{i}}$ **to the first 3 principal components obtained for the Roads set of networks analyzed.**


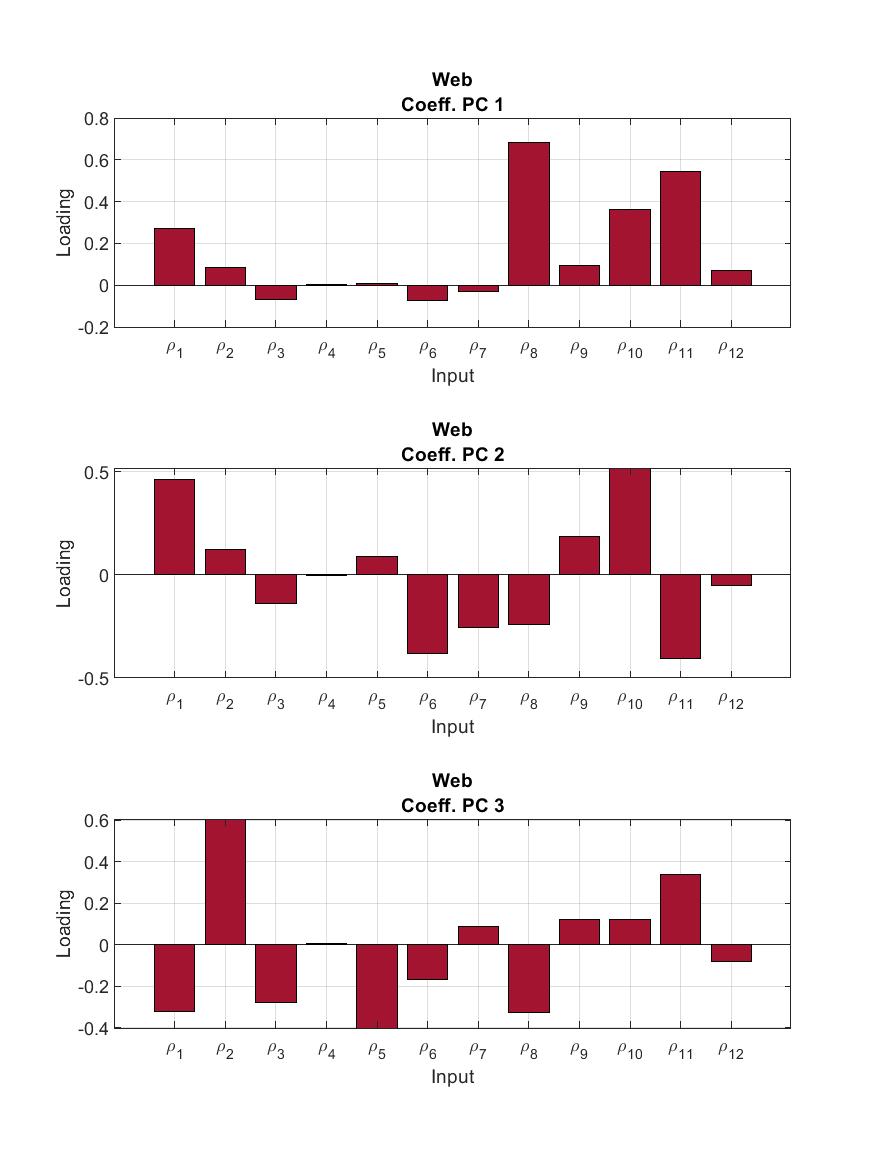


SI-Fig. 10. **Contributions of each component** $\boldsymbol{\rho}_{\boldsymbol{i}}$ **to the first 3 principal components obtained for the Web set of networks analyzed.**

## Range of $\boldsymbol{\rho}$ by type of network

SI-Table 2

Values of $\rho$ for autonomous-system graphs.

|  | $\rho_{1}$ | $\rho_{2}$ | $\rho_{3}$ | $\rho_{4}$ | $\rho_{5}$ | $\rho_{6}$ | $\rho_{7}$ | $\rho_{8}$ | $\rho_{9}$ | $\rho_{10}$ | $\rho_{11}$ | $\rho_{12}$ |
| --- | --- | --- | --- | --- | --- | --- | --- | --- | --- | --- | --- | --- |
| Minimum | 0.409 | 0.397 | 0.172 | 0.001 | 0.686 | 0.384 | 0.044 | 0.003 | 0.842 | 0.272 | 0.228 | 0.005 |
| Quantile 1 | 0.459 | 0.577 | 0.191 | 0.001 | 0.699 | 0.456 | 0.144 | 0.004 | 0.917 | 0.318 | 0.496 | 0.007 |
| Quantile 2 | 0.477 | 0.597 | 0.217 | 0.001 | 0.703 | 0.463 | 0.076 | 0.004 | 0.881 | 0.340 | 0.527 | 0.009 |
| Quantile 3 | 0.510 | 0.619 | 0.257 | 0.002 | 0.762 | 0.431 | 0.087 | 0.010 | 0.884 | 0.369 | 0.430 | 0.016 |
| Maximum | 0.569 | 0.668 | 0.573 | 0.041 | 0.867 | 0.486 | 0.333 | 0.077 | 0.953 | 0.476 | 0.558 | 0.162 |

SI-Table 3

Values of $\rho$ for Enzymes.

|  | $\rho_{1}$ | $\rho_{2}$ | $\rho_{3}$ | $\rho_{4}$ | $\rho_{5}$ | $\rho_{6}$ | $\rho_{7}$ | $\rho_{8}$ | $\rho_{9}$ | $\rho_{10}$ | $\rho_{11}$ | $\rho_{12}$ |
| --- | --- | --- | --- | --- | --- | --- | --- | --- | --- | --- | --- | --- |
| Minimum | 0.113 | 0.000 | 0.111 | 0.101 | 0.342 | 0.000 | 0.000 | 0.000 | 0.000 | 0.000 | 0.000 | 0.400 |
| Quantile 1 | 0.447 | 0.000 | 0.143 | 0.301 | 0.777 | 0.000 | 0.000 | 0.109 | 0.551 | 0.735 | 0.027 | 0.607 |
| Quantile 2 | 0.487 | 0.000 | 0.167 | 0.361 | 0.831 | 0.000 | 0.000 | 0.175 | 0.647 | 0.969 | 0.085 | 0.660 |
| Quantile 3 | 0.524 | 0.000 | 0.200 | 0.428 | 0.873 | 0.052 | 0.000 | 0.274 | 0.709 | 1.000 | 0.156 | 0.720 |
| Maximum | 0.604 | 0.308 | 0.833 | 0.693 | 0.946 | 0.800 | 0.848 | 0.417 | 0.829 | 1.000 | 0.556 | 0.900 |

SI-Table 4

Values of $\rho$ for Facebook graphs.

|  | $\rho_{1}$ | $\rho_{2}$ | $\rho_{3}$ | $\rho_{4}$ | $\rho_{5}$ | $\rho_{6}$ | $\rho_{7}$ | $\rho_{8}$ | $\rho_{9}$ | $\rho_{10}$ | $\rho_{11}$ | $\rho_{12}$ |
| --- | --- | --- | --- | --- | --- | --- | --- | --- | --- | --- | --- | --- |
| Minimum | 0.949 | 0.548 | 0.016 | 0.000 | 0.934 | 0.000 | 0.000 | 0.035 | 0.997 | 0.927 | 0.004 | 0.009 |
| Quantile 1 | 0.970 | 0.623 | 0.051 | 0.002 | 0.982 | 0.006 | 0.000 | 0.050 | 0.998 | 0.955 | 0.010 | 0.039 |
| Quantile 2 | 0.974 | 0.648 | 0.076 | 0.007 | 0.986 | 0.008 | 0.013 | 0.058 | 0.999 | 0.964 | 0.013 | 0.066 |
| Quantile 3 | 0.977 | 0.681 | 0.122 | 0.017 | 0.987 | 0.010 | 0.027 | 0.071 | 0.999 | 0.972 | 0.016 | 0.110 |
| Maximum | 0.983 | 0.838 | 0.269 | 0.069 | 0.992 | 0.018 | 0.080 | 0.120 | 1.000 | 0.988 | 0.044 | 0.224 |

SI-Table 5

Values of $\rho$ for power-network graphs.

|  | $\rho_{1}$ | $\rho_{2}$ | $\rho_{3}$ | $\rho_{4}$ | $\rho_{5}$ | $\rho_{6}$ | $\rho_{7}$ | $\rho_{8}$ | $\rho_{9}$ | $\rho_{10}$ | $\rho_{11}$ | $\rho_{12}$ |
| --- | --- | --- | --- | --- | --- | --- | --- | --- | --- | --- | --- | --- |
| Minimum | 0.127 | 0.060 | 0.185 | 0.030 | 0.512 | 0.190 | 0.067 | 0.017 | 0.056 | 0.117 | 0.097 | 0.233 |
| Quantile 1 | 0.219 | 0.198 | 0.271 | 0.078 | 0.719 | 0.345 | 0.172 | 0.027 | 0.143 | 0.192 | 0.154 | 0.320 |
| Quantile 2 | 0.260 | 0.303 | 0.340 | 0.104 | 0.767 | 0.401 | 0.263 | 0.030 | 0.251 | 0.225 | 0.209 | 0.428 |
| Quantile 3 | 0.285 | 0.382 | 0.446 | 0.196 | 0.799 | 0.426 | 0.339 | 0.037 | 0.380 | 0.297 | 0.252 | 0.532 |
| Maximum | 0.466 | 0.600 | 0.619 | 0.310 | 0.853 | 0.564 | 0.486 | 0.083 | 0.688 | 0.451 | 0.353 | 0.714 |

SI-Table 6

Values of $\rho$ for retweet graphs.

|  | $\rho_{1}$ | $\rho_{2}$ | $\rho_{3}$ | $\rho_{4}$ | $\rho_{5}$ | $\rho_{6}$ | $\rho_{7}$ | $\rho_{8}$ | $\rho_{9}$ | $\rho_{10}$ | $\rho_{11}$ | $\rho_{12}$ |
| --- | --- | --- | --- | --- | --- | --- | --- | --- | --- | --- | --- | --- |
| Minimum | 0.014 | 0.609 | 0.132 | 0.001 | 0.244 | 0.293 | 0.000 | 0.000 | 0.000 | 0.001 | 0.057 | 0.004 |
| Quantile 1 | 0.065 | 0.730 | 0.209 | 0.002 | 0.408 | 0.439 | 0.050 | 0.000 | 0.463 | 0.015 | 0.161 | 0.019 |
| Quantile 2 | 0.127 | 0.810 | 0.327 | 0.003 | 0.506 | 0.483 | 0.084 | 0.000 | 0.538 | 0.027 | 0.237 | 0.041 |
| Quantile 3 | 0.182 | 0.902 | 0.575 | 0.004 | 0.621 | 0.618 | 0.118 | 0.001 | 0.678 | 0.048 | 0.476 | 0.069 |
| Maximum | 0.616 | 0.968 | 0.926 | 0.086 | 0.838 | 0.795 | 0.194 | 0.026 | 0.959 | 0.202 | 0.722 | 0.347 |

SI-Table 7

Values of $\rho$ for roads graphs.

|  | $\rho_{1}$ | $\rho_{2}$ | $\rho_{3}$ | $\rho_{4}$ | $\rho_{5}$ | $\rho_{6}$ | $\rho_{7}$ | $\rho_{8}$ | $\rho_{9}$ | $\rho_{10}$ | $\rho_{11}$ | $\rho_{12}$ |
| --- | --- | --- | --- | --- | --- | --- | --- | --- | --- | --- | --- | --- |
| Minimum | 0.042 | 0.019 | 0.119 | 0.018 | 0.723 | 0.098 | 0.186 | 0.000 | 0.000 | 0.002 | 0.006 | 0.242 |
| Quantile 1 | 0.065 | 0.035 | 0.182 | 0.048 | 0.766 | 0.121 | 0.224 | 0.001 | 0.026 | 0.003 | 0.018 | 0.311 |
| Quantile 2 | 0.185 | 0.058 | 0.207 | 0.066 | 0.801 | 0.570 | 0.405 | 0.009 | 0.041 | 0.055 | 0.021 | 0.378 |
| Quantile 3 | 0.284 | 0.196 | 0.246 | 0.102 | 0.842 | 0.816 | 0.770 | 0.021 | 0.102 | 0.162 | 0.044 | 0.464 |
| Maximum | 0.294 | 0.217 | 0.371 | 0.218 | 0.846 | 0.889 | 0.882 | 0.021 | 0.121 | 0.165 | 0.096 | 0.683 |

SI-Table 8

Values of $\rho$ for web graphs.

|  | $\rho_{1}$ | $\rho_{2}$ | $\rho_{3}$ | $\rho_{4}$ | $\rho_{5}$ | $\rho_{6}$ | $\rho_{7}$ | $\rho_{8}$ | $\rho_{9}$ | $\rho_{10}$ | $\rho_{11}$ | $\rho_{12}$ |
| --- | --- | --- | --- | --- | --- | --- | --- | --- | --- | --- | --- | --- |
| Minimum | 0.369 | 0.497 | 0.012 | 0.000 | 0.555 | 0.029 | 0.000 | 0.000 | 0.689 | 0.218 | 0.069 | 0.001 |
| Quantile 1 | 0.532 | 0.608 | 0.022 | 0.000 | 0.672 | 0.075 | 0.032 | 0.017 | 0.916 | 0.380 | 0.172 | 0.003 |
| Quantile 2 | 0.701 | 0.687 | 0.146 | 0.001 | 0.735 | 0.185 | 0.059 | 0.060 | 0.984 | 0.591 | 0.229 | 0.045 |
| Quantile 3 | 0.872 | 0.802 | 0.247 | 0.007 | 0.800 | 0.243 | 0.116 | 0.275 | 0.995 | 0.739 | 0.554 | 0.066 |
| Maximum | 0.989 | 0.910 | 0.481 | 0.023 | 0.938 | 0.352 | 0.413 | 0.999 | 1.000 | 0.989 | 0.984 | 0.216 |

## Comparison with other dimensionality reduction techniques

In order to validate the principal component analysis, two additional dimensionality reduction techniques have been applied to the network dataset. This section summarizes the results of each method and compare them with the results included in the paper.

### Independent Component Analysis

Firstly, a Independent Component Analysis (ICA) is performed. Unlike PCA, which tries to maximize the uncorrelatedness of the principal components, ICA aims at maximizing the independence between each reduced component. In order to fit this model, R’s fastICA library was used. The results of this model can be observed in SI-Fig. 11 and SI-Fig. 12, which illustrate the 3-dimensional representation after projecting the original dataset into the three first independent components.

Similarly to the PCA, there are four clearly identified clusters, associated with four different types of networks: Enzymes, Facebook, Autonomous Systems and Retweet. However, this representation highlights the heterogeneity of the Web networks, as they appear more scattered than in the PCA representation. Regarding Power Network and Roads networks, they exhibit similar features than the ones shown in the PCA study.


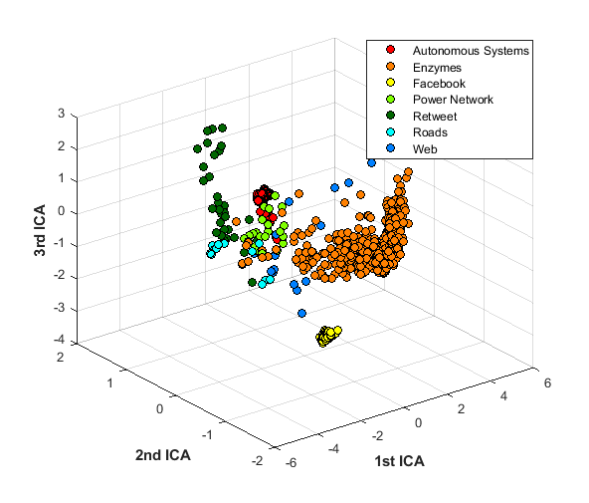


SI-Fig. 11. **Graphical representation of the 1404 networks in the 3-d space defined by the first three independent components.**


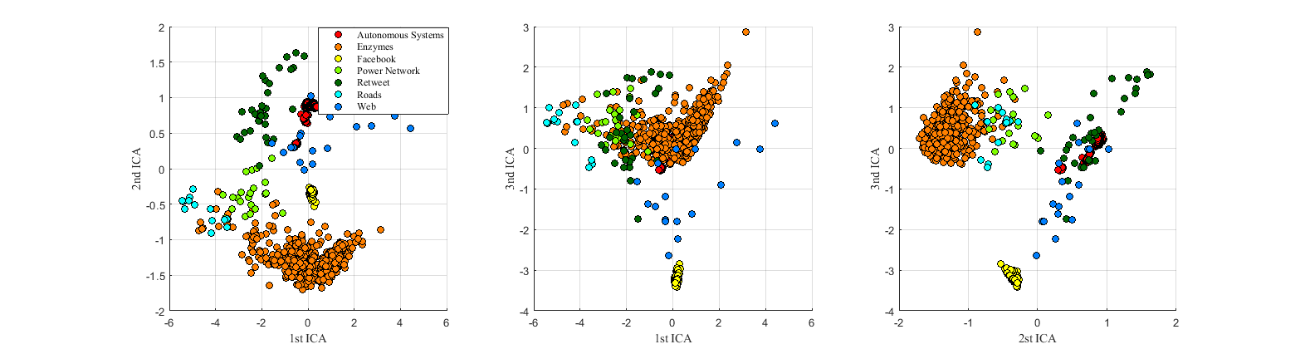


SI-Fig. 12. **2-d projections of the 3-d representation of the 1404 networks in the space defined by the three first independent components.**

### Self-Organizing Map

The second dimensionality reduction technique used is the Self-Organizing Map (SOM). This technique tries to find a low-dimensional representation of the data in such a way that the topological ordering properties of the original data are preserved. This model was fitted using R’s kohonen library, and the particular configuration of the SOM network was a $10\times10$ hexagonal grid.

In SI-Fig. 13 the centers of the fitted network are plotted. Any point that belongs to a given center must exhibit similar values of the twelve-dimensional metric proposed. For example, points that belong to the bottom-right center will have high values of ${\rho_{1},\rho_{2},\rho}_{4}, \rho_{9}$ and $\rho_{10}$ and very low values of the other components. This allows for an easier interpretation of the results of the model, as well as a straightforward method to cluster the original data: a hierarchical clustering of the centers was performed, obtaining five different clusters. Centers belonging to the same cluster will show similar properties.

SI-Fig. 13. **Centers of each cell of the SOM. Thicker lines indicate the 5 different clusters that were obtained. Higher values of each dimension are represented by a great colored area.**

Once the SOM model was fitted, each network was projected in this low-dimensional representation, as SI-Fig. 13 illustrates. In this representation, cells that belong to the same cluster appear in the same color. By examining each cluster, parallels can be drawn with the PCA study: again, Enzymes networks appear in one clearly defined cluster, characterized by high values of $\rho_{5},\rho_{10}$ and $\rho_{12}$ and great variability among its elements. In a similar way, Autonomous Systems appear in their own cluster, characterized by high values of $\rho_{9}$ and $\rho_{5}.$ Facebook and Retweet networks also appear in very differenced clusters, as was observed in the principal component analysis. However, the representation of the Web network difers from the PCA representation: in the SOM projection, Web’s networks are scattered across several clusters, showing the heterogeneity of the topological structure of these networks. Despite this, SOM’s classification retains certain relevant properties first seen in the PCA: the nearest networks for the Web are again Autonomous Systems and Facebook.

SI-Fig. 14. **Projections of the networks into the SOM representation. Each point represents one network, and each cell is colored according to its cluster.**

## References

1. Watts, D. J. & Strogatz, S. H. Collective dynamics of ‘small-world’ networks. Nature 393, 440–442 (1998).

2. Rossi, R. & Ahmed, N. The Network Data Repository with Interactive Graph Analytics and Visualization. in Twenty-Ninth AAAI Conference on Artificial Intelligence (2015).

3. Guimerà, R., Danon, L., Díaz-Guilera, A., Giralt, F. & Arenas, A. The real communication network behind the formal chart: Community structure in organizations. Journal of Economic Behavior & Organization 61, 653–667 (2006).

4. Schellenberger, J., Park, J. O., Conrad, T. M. & Palsson, B. Ø. BiGG: a Biochemical Genetic and Genomic knowledgebase of large scale metabolic reconstructions. BMC Bioinformatics 11, 213 (2010).

5. Ebel, H., Mielsch, L.-I. & Bornholdt, S. Scale-free topology of e-mail networks. Phys. Rev. E 66, 035103 (2002).
